# Controllable gelation of artificial extracellular matrix for altering mass transport and improving cancer therapies

**DOI:** 10.1038/s41467-020-18493-7

**Published:** 2020-09-30

**Authors:** Di-Wei Zheng, Sheng Hong, Qiu-Ling Zhang, Xue Dong, Pei Pan, Wen-Fang Song, Wen Song, Si-Xue Cheng, Xian-Zheng Zhang

**Affiliations:** Key Laboratory of Biomedical Polymers of Ministry of Education & Department of Chemistry, Wuhan University, Wuhan, 430072 P.R. China

**Keywords:** Cancer therapy, Biomedical materials

## Abstract

Global alterations in the metabolic network provide substances and energy to support tumor progression. To fuel these metabolic processes, extracellular matrix (ECM) plays a dominant role in supporting the mass transport and providing essential nutrients. Here, we report a fibrinogen and thrombin based coagulation system to construct an artificial ECM (aECM) for selectively cutting-off the tumor metabolic flux. Once a micro-wound is induced, a cascaded gelation of aECM can be triggered to besiege the tumor. Studies on cell behaviors and metabolomics reveal that aECM cuts off the mass transport and leads to a tumor specific starvation to inhibit tumor growth. In orthotopic and spontaneous murine tumor models, this physical barrier also hinders cancer cells from distant metastasis. The in vivo gelation provides an efficient approach to selectively alter the tumor mass transport. This strategy results in a 77% suppression of tumor growth. Most importantly, the gelation of aECM can be induced by clinical operations such as ultrasonic treatment, surgery or radiotherapy, implying this strategy is potential to be translated into a clinical combination regimen.

## Abstract (web summary)

The extracellular matrix (ECM) can influence tumor growth and its response to therapy. Here, the authors develop a fibrinogen and thrombin based artificial ECM that can starve tumours and prevent dissemination of cancer cells.

## Introduction

The reprogram of metabolism is a characteristic phenotype of cancers^1^. Rewiring of metabolism pathways, such as glucose metabolism, urea cycle metabolism, and lipid metabolism, maximizes the use of nitrogen/carbon sources for macromolecular metabolic synthesis^2^. As an efficient anti-cancer strategy, targeting certain metabolism pathways has achieved some success^3^. For example, l-asparaginase (for glutamine depletion) and 3-bromopyruvic acid (for glycolysis inhibition) have successfully passed clinical trials^4^. However, the tumor metabolism is a network of countless reactions. Under the selective pressure of molecular targeted therapy, drug resistance mutations occur commonly^5^. High-dose toxicity and drug resistance greatly plague the clinical effect of these therapies. Rather than focusing on one single pathway, finding strategies from a systematic perspective should be promising.

As one of the most important non-cellular components in tumor microenvironment, extracellular matrix (ECM) is the structural foundation and biochemical support for tumors^6^. For its abnormal metabolic network, the tumor tissue is vastly different from normal tissues in terms of nutrient intake and waste accumulation. As an efficient mass transport medium, ECM is critical to satisfy the need of cancer cells in maintaining rapid growth and continuous proliferation^7^. Changes in the ECM structure are known to elicit circumscribed metabolic responses, as the decrease of hyaluronidases in ECM has been reported to trigger a robust increase in glycolysis^8^. The normalization of the tumor ECM with pharmaceutical agents such as losartan has already shown to improve tumor perfusion^9^. Encouraged by this process, it is promising to induce chemical reactions in ECM for cutting off the nutrition supply and improving tumor perfusion at the same time.

Through ingenious designs and versatile functionalizations, reactive materials have been developed to regulate the tumor metabolism. Nanoparticles such as Mg_2_Si nano-aggregates, BN nano-spheres, and DNA origami nano-carriers have been synthesized to cut off the energy supply of tumors^10–12^. Besides, the concept of biomedical photocatalysis has also been proposed to controllably produce gaseous signal molecules (CO and NO) for adjusting tumor redox equilibrium^13–16^. In a large number of pre-clinical experiments, the efficacy of these materials in sensitizing conventional therapies (e.g. chemotherapy, radiotherapy (RT), and phototherapy) has been proved^17–19^. These achievements have initiated the idea of using reactive materials to modulate the complex biological characteristics of tumors.

**Fig. 1:**
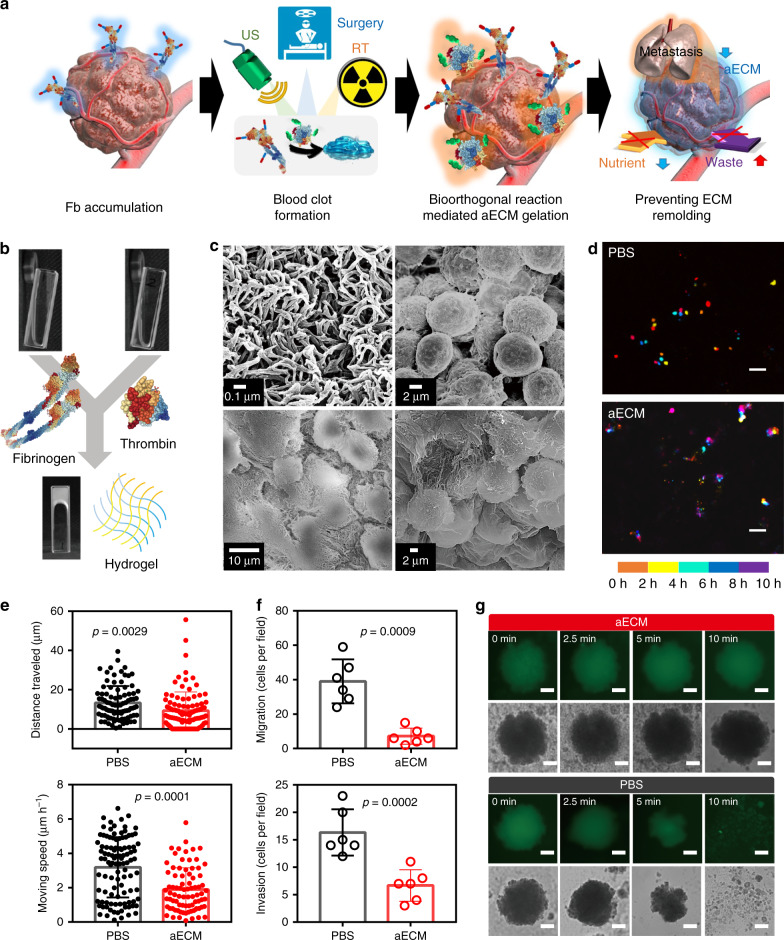
In vitro gelation of aECM. **a** Schematic diagram of the formation of aECM. A small-scaled gelation was induced by US, surgery, or radiotherapy. The tumor-specific accumulation of Ptb-DBCO was guided by the bioorthogonal reaction between DBCO and N_3_ groups. The second US treatment was used to induce the large-scaled gelation. **b** In vitro gelation of fibrinogen with thrombin. **c** SEM of fibrin hydrogel and CT26 cells encapsulated in fibrin hydrogel. The experiment was repeated twice independently with similar results. **d** Cell migration study of CT26 cells with or without aECM treatment (*n* = 4 fields). Positions of cells at different time-points were pseudocoloured. The continuous culture apparatus was used for long-term observations (scale bar: 50 μm). **e** Quantitative analysis for the cell migration study. Moving speeds and squared displacement values of CT26 cells were calculated from ~100 cells. The results were calculated with Image J plug-in, MTrack2 (up: PBS *n* = 93, aECM *n* = 100; down: PBS *n* = 105, aECM *n* = 81). **f** Transwell inserts for migration and invasion detections (*n* = 6 fields). After 48 h incubation, the cell numbers in down-chambers were counted. The number of cells in the 200-fold magnified field of view was counted. **g** Images of CT26 MTS with and without aECM treatment under shaken (300 r.p.m.) for 10 min. A representative image of three biological replicates is shown (scale bar: 100 μm). Significance between two groups (**e**, **f**) was calculated by using two-tailed Student’s *t-*test. The mean values and SD are presented.

In response to bleeding, the fibrinogen in plasma is proteolytically activated by thrombin to form gel-like clots. Inspired by this, we successfully engineered two US-FDA-approved pharmaceuticals to obtain azido-modified fibrinogen (Fb-N_3_) and azodibenzocyclooctyne (DBCO)-grafted prothrombin (Ptb-DBCO), respectively. As shown in Fig. 1a, after intravenous injection (i.v.) of Fb-N_3_, its tumor-specific accumulation could be triggered by micro-wound made through surgery, RT, or ultrasonic (US) treatment^20–22^. Subsequently, the intravenously injected Ptb-DBCO was accumulated through bioorthogonal reaction between DBCO and N_3_ groups^23^. Owing to the altered vascular structure, we expect that the accumulated Ptb would easily translate into active enzyme (thrombin) and induce coagulation. The retiform clots besiege the tumor, resulting in an aECM fibrin gel for blocking nutrient exchange and preventing of tumor cell migration. This controllable gelation strategy can regulate metabolism of tumors and synergize with surgery, RT, chemotherapy, or even immunotherapy.

## Results

### In vitro gelation of aECM

As shown in Fig. 1b, the fibrinogen could be transformed into a fibrin hydrogel with the addition of thrombin in a few minutes. The transformation was also confirmed by ultraviolet–visible spectrum (Supplementary Fig. 1). The hydrogel microstructures were observed by scanning electron microscopy (SEM) (Fig. 1c), which shows the formation of hydrogel from nano-fibers with a complex 3D structure. The gelation was also used to trap CT26 cells. SEM images confirm that CT26 cells could be completely encapsulated in a dense fibrous network.

Cell migration is the very first step for tumor metastasis. Here, we investigated the effect of aECM gelation on cell migration. Results from in situ dynamic tracing showed that the migration capacity of cancer cells dropped dramatically after the aECM gelation (Fig. 1d and Supplementary Fig. 2)^24^. The speed and squared displacement values of cell migration were calculated. Quantitatively, the aECM encapsulation reduced the movement speed of cells by 41% (Fig. 1e). To further confirm the anti-migration ability of aECM, transwell migration assay was performed. The cells in the control group displayed longer migration distances as compared with those in the aECM group (Fig. 1f). The invasion of CT26 cells was also studied with collagen-coated Transwell chambers. It was found that the aECM treatment could inhibit the invasion of CT26 cells by 59.2% (Fig. 1f). In order to partly simulate the physiological features of solid tumor in vitro, we established a multicellular tumor spheroids (MTS) model (Fig. 1g)^25^. After shaking at 300 r.p.m. for 10 min, MTS in control groups were broken into small fragments, while no obvious changes could be observed in the aECM group. In consideration of these results, we deduce that aECM gelation might prevent cancer cells from moving for distant metastasis.

### Anti-cancer mechanism of aECM

**Fig. 2:**
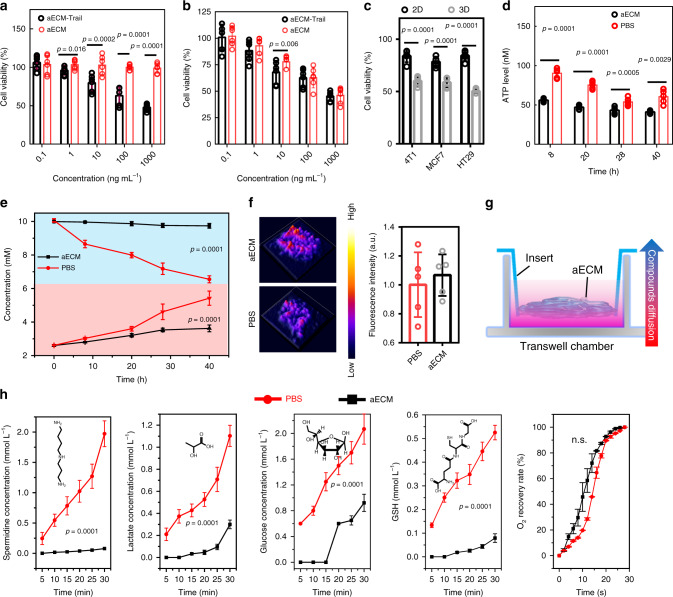
Anti-cancer mechanism of aECM. **a** Cytotoxicity of different concentrations of aECM or aECM-Trail (0.1–1000 ng mL^−1^) towards CT26 cells. Six biological replicates are shown. **b** Cytotoxicity of different concentrations of aECM or aECM-Trail (0.1–1000 ng mL^−1^) towards CT26 MTS. CT26 MTS were treated with aECM or aECM-Trail for 24 h. Six biological replicates are shown. **c** Cytotoxicity of aECM towards 2D cell culture system and 3D MTS model in 4T1, MCF-7, and HT-29 cells for 24 h. Five biological replicates are shown. **d** ATP level in CT26 MTS after being treated with aECM or PBS. The ATP levels were measured at the different time points during the treatment. Five biological replicates are shown. **e** Real-time glucose and lactate concentrations in the culture medium of aECM or PBS treated CT26 MTS. Six biological replicates are shown. The upper curves represent glucose, and the lower curves represent lactic acid. **f** Oxygen content in CT26 MTS after being treated with PBS or aECM. ROS-ID was used to visualize the hypoxia levels in CT26 MTS. Five biological replicates are shown. **g** Schematic diagram of the diffusion assay based on Transwell insert. A high concentration solution was added in the lower chamber and DI water was placed in the upper chamber. aECM or collagen was coated on the porous membrane. **h** The effect of aECM coating on suppressing mass transports (spermidine, lactate, glucose, glutathione, and O_2_). Collagen-coated Transwell inserts were set as a control (black). Three biological replicates are shown. Significance between two groups (**a**–**f**, **h**) was calculated by using two-tailed Student’s *t-*test. The mean values and SD are presented.

Initially, to enhance the anti-cancer capacity of aECM, tumor necrosis factor-related apoptosis-inducing ligand (Trail) was conjugated to aECM (aECM-Trail)^26^. The influence of aECM and aECM-Trail on cell proliferation was studied in both 2D cell culture system and 3D MTS model. In the 2D cell culture system, aECM-Trail displayed obvious cytotoxicity towards CT26 cells, and about 52.1% of cancer cells were eliminated at a concentration of 1000 ng mL^−1^ (Fig. 2a, b and Supplementary Fig. 3). Unexpectedly, it was observed that both aECM and aECM-Trail could inhibit the proliferation of CT26 cells in the 3D MTS model. In total, 54.0% and 54.7% of cancer cells were eliminated by aECM and aECM-Trail at a concentration of 1000 ng mL^−1^, respectively. The same phenomenon was also observed in MTS models of 4T1, MCF-7, and HT29 cells (Fig. 2c). Interestingly, by using ATP bioluminescence assay, it was noticed that aECM treatment could markedly reduce the intracellular ATP level in the 3D MTS model (Fig. 2d). Thus, we speculate that the inhibition of CT26 cells growth in the 3D MTS model might be attributed to the suppressed cellular metabolism. To prove this conjecture, both glucose and lactate levels in CT26 MTS were measured. After aECM treatment, significant reductions of glucose consumption and lactate production were found (Fig. 2e). From these results, it was concluded that aECM treatment could suppress the cell respiration in MTS. The dense structure in hydrogels might restrict molecular diffusion, thus triggering cellular starvation by cutting off the nutrition intake of cancer cells^27^. It has been previously reported that the hypoxia caused by starvation therapy not only led to the upregulation of angiogenic factor within the tumor, but also triggered the tumor metastasis^28^. In this research, by using ROS-ID probe, we noticed that aECM encapsulation did not aggravate cellular hypoxia (Fig. 2f). Commonly, hypoxia induces angiogenesis to accelerate the tumor growth. Thus, it could be deduced that aECM might have permselectivity for different molecules.

To validate this conjecture, we designed a diffusion chamber. Condensed solution was added to the lower chamber and DI water was placed in the upper chamber, leading to a sharp concentration gradient between two chambers (Fig. 2g). Here, aECM acted as a membrane to separate these two chambers. First, the influence of aECM on the diffusion of five typical metabolic molecules, including spermidine, lactate, glucose, glutathione (GSH), and O_2_, was studied. As shown in Fig. 2h, within 0.5 h, only 3% of spermidine entered the upper chamber. In contrast, 25% of lactate and 18% of GSH diffused through the hydrogel and reached the upper chamber. However, aECM did not show any steric hindrance towards the gaseous molecules—the dissolved O_2_.

### Selective permeability of aECM

**Fig. 3:**
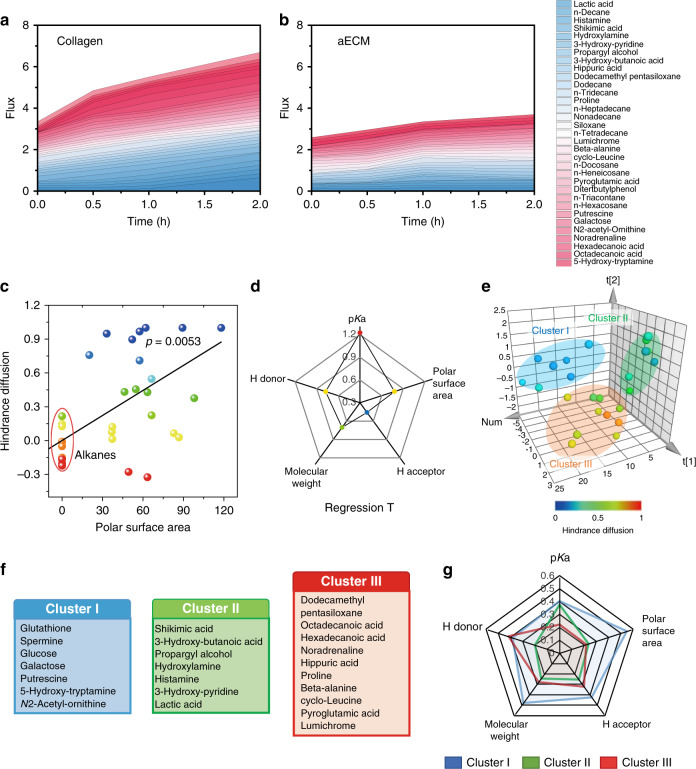
In vitro selective permeability of aECM. **a**, **b** Diffusion of 33 serum metabolites through collagen-coated or aECM-coated membrane at different time points. The proportion of molecules that diffused through the membrane was recorded. The average value of three biological replicates is shown. **c** Linear-regression analysis of polar surface area to hindrance diffusion. Non-polar molecules (circled in red) could easily pass through aECM gel. The larger the polar surface area, the more difficult it was for the molecules to diffuse through aECM gel. **d** Regression *T-*test summary for analyzing the most important molecular structural factors that influence diffusion. **e** Principal component analysis for clustering 33 metabolites into three independent groups. Compounds in different clusters have certain characteristics in the molecular structure. Data points were pseudocoloured to represent the hindrance diffusion. **f** The lists of metabolites in cluster I, cluster II, and cluster III. **g** Radar plot for illustrating the contribution of p*K*a, H donor, molecule weight, H acceptor, and polar surface area to the grouping of each molecule. The higher the *F*-factor, the greater the influence of physicochemical property towards molecular diffusion.

We further investigated the influence of aECM on the diffusion of main components in serum. In this study, molecules diffused through the aECM or collagen (main component of ECM) membrane were determined and measured with a gas chromatography-mass spectrometer (GC/MS). Within 2 h, aECM was found to prevent 34% of small molecules from transporting through the membrane as compared with the control group (Fig. 3a, b). We also observed that the rate of molecular diffusion was dependent on the molecular structure. Long-chain alkanes such as docosane and dodecane could easily pass through aECM. To prove this, a scatter diagram of hindrance diffusion versus polar surface area was performed. Fig. 3c shows that the hindrance of diffusion increases with increasing molecular polarity of an another repeated experiment. In term of polar surface area, it is clear that the alkanes display extremely lower diffusion hindrance compared with other compounds.

However, it should be noted that only one variable could not comprehensively explain the selective permeability of aECM. Furthermore, the molecular weight, H acceptor, H donor, polar surface area, and p*K*a of these molecules were obtained from Scifinder database, and a linear regression analysis was also performed. The weight of each variable was calculated and their T factors are displayed in a radar chart (Fig. 3d). Here, p*K*a, the polar surface area, and H donor exhibited relative strong linear relationship with the diffusion rate (*F*  > 0.5). However, the significant correlations of the molecule weight/H acceptor with the diffusion rate were also noticed (Supplementary Fig. 4). Overall, it can be concluded that the diffusion capacity of these molecules is multifactorial. Afterwards, a multivariate analysis (principal component analysis, PCA) was performed. In this way, 33 small molecules could be classified by assessing the proximity of the PCA points. All these compounds were divided into three non-overlapping clusters (Fig. 3e). Strikingly, all molecules in cluster I could hardly permeate through the aECM membrane (Fig. 3f). In contrast, molecules in cluster III exhibited the strongest diffusion capacity, and molecules in cluster II were in between. Furthermore, structural features of molecules in three clusters were analyzed. The radar plot in Fig. 3g demonstrates that molecules in different clusters display different molecular characteristics. It could be observed that molecules with the strong polarity, low p*K*a and high molecular weight were inclined to be obstructed by aECM. However, molecules with the high p*K*a and weak polarity could pass through the membrane with low resistance.

Next, both grand average of hydropathicity index (GRAVY) and isoelectric point (*pI*) of fibrinogen were calculated by ExPASy ProtParam^29^. The GRAVY of fibrinogen was determined to be −0.577, indicating its well-solubility. Hydrophilic molecules in cluster I tended to form hydrogen bonds with amide bonds of aECM. In contrast, more hydrophobic molecules in cluster III such as long-chain fatty acids could easily penetrate through the aECM membrane. The *pI* value of fibrinogen was calculated to be 5.56. The strong electrostatic interaction between aECM and molecules in cluster II (with low p*K*a) also prevented the diffusion.

### In vivo metabolism study

**Fig. 4:**
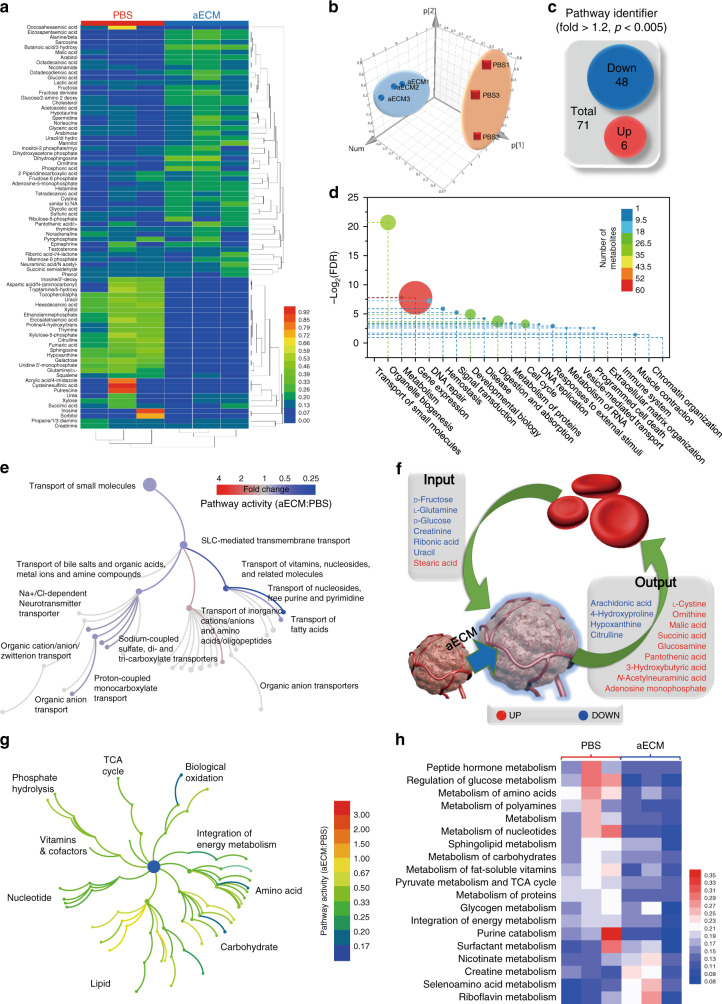
In vivo metabonomics analysis of aECM-treated tumors. **a** Heat map representation and cluster analysis of metabolites in mice tumors after treated with aECM or PBS. Three biological replicates are shown. **b** Principal component analysis of metabolites in mice CT26 tumors after treated with aECM or PBS. **c** Venn diagram of differential metabolites (fold change > 1.2; hypergeometric test *p* < 0.005) between aECM and PBS-treated tumors. **d** Bubble diagram of differential metabolites that enriched in various Reactome pathways. Circle diameters represent the number of metabolites in each pathway. Circles were pseudocoloured to show the number of metabolites in each pathways. **e** The influence of aECM treatment towards pathways related to “SLC-mediated transmembrane transport process”. Topological graph of these pathways are shown. Metabolomics data were analyzed with Reactome database. Circles were pseudocoloured to represent changes of pathway activity, and circle diameters represent the number of metabolites in each pathway. **f** The list of input and output metabolites of tumors. aECM treatment suppressed the input of nutrients and inhibited the output of wastes. **g** Topological graph of “cell metabolism process” annotated in the Reactome database. **h** Heat map of significantly enriched pathways in “cell metabolism process” (false discovery rate < 0.05) before and after aECM treatment. Three biological replicates are shown. Significance between two groups (**a, h**) was calculated by using two-tailed Student’s *t-*test.

It was speculated that the intratumoral formation of aECM might lead to systemic metabolic changes. Here, metabolomics analysis was performed on tumors with or without aECM treatments. In total, 146 metabolites were identified with GC-MS analysis (Fig. 4a). Custer analysis was performed on all these samples, which could be classified into two unsupervised horizontal clusters. Then, PCA analysis was used to determine the influence of prognostic variables (metabolite content). In general, this result indicated that the metabolism of aECM-treated CT26 tumors was highly distinct from that of control groups (Fig. 4b). Most of the down-regulated molecules had strong polarity, low p*K*a, and high molecular weight. This result reminded us that change of tumor metabolism might correlate to the selective permeability of aECM. This conjecture was also supported by linear-regression analysis and PCA analysis (Supplementary Fig. 5)^30^.

Metabolomic data were analyzed with the Reactome database. After the annotation, it was found that most pathways (48 of 71) were inhibited after the gelation, and only 8% of pathways were upregulated (Fig. 4c). Among all these pathways, both metabolism processes and translation of small molecules showed low false discovery rate (FDR) values and major changes. It was noticed that aECM encapsulation treatment affected 36.7% of reactions (found: 143; total: 390) in the pathway of small molecules translation. At the same time, in the network of metabolism, 32.1% of reactions (found: 670; total: 2086) were influenced (Fig. 4d).

Specifically, for small molecules, the transport of vitamins, nucleosides, carboxylates, and organic anions was suppressed. Both fatty acids and organic anions transports were not largely affected (Fig. 4e). The result of in vivo experiment was also in agreement with the previous diffusion experiment. Subsequently, different metabolites were classified into two groups. As shown in Fig. 4f, the decrease of input nutrients such as stearic acid, uracil, ribonic acid, creatinine, d-glucose, l-glutamine, and d-fuctose could be observed. In contrast, intratumoral accumulation of metabolic wastes, including adenosine monophosphate, acetylneuraminic acid, hydroxybutyric acid, pantothenic acid, glucosamine acid, succinic acid, malic acid, ornithine, and l-cystine was noticed. Obviously, this result also demonstrates that aECM treatment could suppress tumor metabolism by limiting nutrient availability and waste discharge. This speculation was further confirmed by Reactome database analysis (Fig. 4g). From this analysis, aECM gelation was found to inhibit the tumor metabolism in a global perspective. Significantly decreased activities of pathways in main metabolic processes such as carbohydrate metabolism, amino acid metabolism, nucleotide metabolism, and lipid metabolism were collectively observed. Furthermore, significant changes in 19 identified pathways listed in a heat map show the overwhelming majority of them (15 of 19 pathways) are inhibited (Fig. 4h). Above all, we hypothesized that aECM might be able to suppress the tumor growth by cutting off the nutrition supply.

### In vivo gelation of aECM

Surgery, RT, and US treatments were carried out to cause micro-wounds and induce coagulation. During this cascade, the enzymatic activation of prothrombin by activated Factor X produced thrombin. Thrombin then attacked α- and β-chains of Fb-N_3_ to form a single fibrin-N_3_ chain. Fbrin-N_3_ strands polymerized and crosslinked with other fibrin-N_3_ scaffolds to form a network structure in the tumor. Data from in vivo fluorescence imaging show that the local treatment of tumor with surgery, RT, or US is efficient in inducing tumor-specific accumulation of Cy7-Fb-N_3_ (Supplementary Fig. 6a–d). In order to assess the in vivo gelation of aECM, in vivo fluorescence imaging system was used. Cyanine7-labeled Fb-N_3_ (Cy7-Fb-N_3_) was intravenously injected into tumor-bearing mice and circulated in the blood. Next, Ptb-DBCO was also injected intravenously into mice. Bioorthogonal reaction between DBCO and N_3_ guided the intratumoral accumulation of Ptb-DBCO. The result from the in vivo fluorescence imaging also revealed the tumor-specific accumulation of Cy7-Ptb-DBCO (Supplementary Fig. 6e). A second US treatment was performed to active Ptb and induce a large-scale gelation. By using thrombin activity fluorometric assay kit, we noticed that the second US treatment amplified the coagulation reaction. Significant fibrin aggregation after the second US treatment was also proved with the in vivo fluorescence imaging and fibrinogen ELISA kit (Supplementary Fig. 6f). Furthermore, the gelation process was also visualized in mouse ears with intradermal tumors by intravital fluorescence microscopy^31^. After the US treatment; it was observed that the rhodamine-labeled Fb was clotted in ear vessels (Supplementary Fig. 7).

To prove whether the aECM therapy could be also applicable to other tumor types, the biological effect of aECM was also verified in 4T1 tumor-bearing mice (Supplementary Fig. 8).

**Fig. 5:**
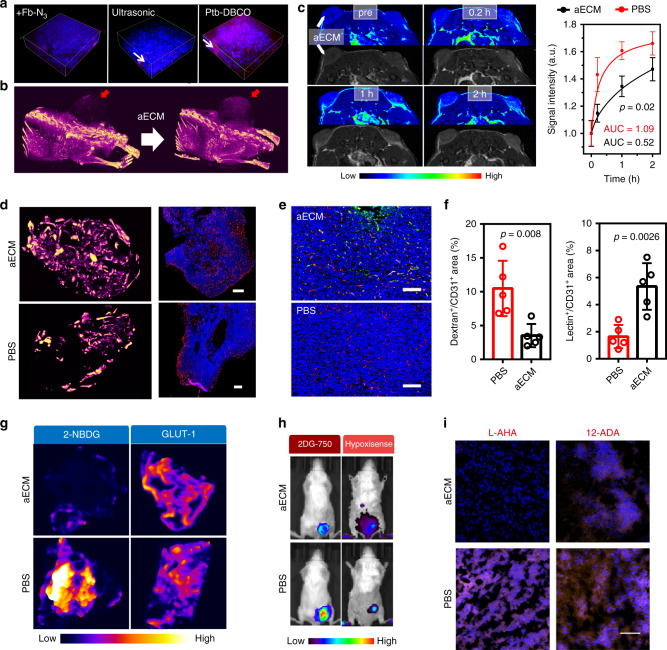
In vivo biological activity of aECM. **a** 3D tumor fluorescence imaging before and after aECM treatment (red: rhodamine B isothiocyanate-labeled fibrinogen; blue: Hochest 33342-strained cells). A representative image of three biological replicates is shown. White arrows indicate the aECM gelation. **b** µ-CT imaging of mice before and after treatment with diatrizoic acid-conjugated aECM. A representative image of three biological replicates is shown. Red arrows denote aECM gelation. **c** The T1-weighted MRI imaging was performed on bilateral tumor-bearing mice, and the representative images and quantitative results of tumors treatment with PBS (right) or aECM (left) are presented. A representative image of three biological replicates is shown. Arrows denote tumor positions. **d** μ-CT imaging for visualizing the three-dimensional structure of tumor vessels. Tumor sections were stained for CD31 (scale bar: 500 μm). A representative image of three biological replicates is shown. **e** CD31 and α-SMA immunostaining of tumor vessels. Tumor sections were stained for CD31 (red) and α-SMA (green) (scale bar: 200 μm). Five images per group were taken. **f** Quantitative analysis of dextran leakage and lectin perfusion of tumor vessels. The tumor sections were stained for CD31 (red). Dextran+ or lectin+ area is presented as a percentage per total sectional or CD31+ area (*n* = 5 fields). **g** The effect of aECM in suppressing glucose uptake and glycolysis. **h** In vivo fluorescence imaging of 2-DG 750 (100 μL) and HypoxiSense 680 (100 μL) for indicating the performances of aECM in affecting tumor glucose uptake and hypoxia. A representative image of three biological replicates is shown. **i** The effect of aECM in suppressing lipid and amino acid uptake. Tumors of 12-ADA- or L-AHA-treated mice were collected 24 h later, and stained with DBCO-Cy5 (*n* = 5 fields, scale bar: 100 μm). Significance between two groups (**c**, **f**) was calculated by using two-tailed Student’s *t-*test. The mean values and SD are presented.

Furthermore, the tumor-specific clotting cascade was verified by tissue clearing and 3D tumor fluorescence imaging technology. After the US treatment, the intense red fluorescence of aECM was observed in 3D tumor fluorescence images (Fig. 5a). Images of Fig. 5b also reveal the enhanced μ-CT signal in the tumor after the treatment with the US-induced aECM gelation.

To estimate the diffusion of small molecules in tumor vasculature after aECM gelation, gadopentetate dimeglumine (Gd-DTPA) was used as the T1-contrast agent for the bilateral CT26 tumor-bearing mice imaging. The right tumor injected with PBS quickly brightened in 10 min, while a negligible signal enhancement in the aECM-treated left tumor was observed (Fig. 5c). This result indicated that the insoluble fibrin formed in situ dramatically diminished the permeability of small molecules through the tumor vasculature.

Subsequently, the biocompatibility and stability of aECM were also evaluated. In blood biochemistry test, hemolysis assay and hematologic analysis, no obvious side effect of aECM gelation could be found (Supplementary Fig. 9). These results indicated the high biocompatibility of aECM^32^.

As a protein, the specificity of aECM makes it possible to be hydrolyzed by proteases (e.g. metalloproteinases MMPs) in the tumor tissues. Thus, the longevity of aECM was estimated (Supplementary Fig. 10). aECM was labeled with the green fluorescent FITC for measuring its in vitro degradation. However, during a 2-week experiment, only 12% of the gel was degraded. Subsequently, Cy7 labeled aECM was implanted into subcutaneous CT26 tumors. No obvious decrease of Cy7 fluorescent signal was found in a 10-day degradation experiment (Supplementary Fig. 10). Additionally, through the immunofluorescence observation, it could also be observed that the degradation of aECM was very slight. The presence of chemical crosslinks between DBCO and N_3_ may be responsible for the difficulty of degradation. The slow degradation characteristics revealed that aECM was promising for in vivo applications.

### In vivo biological effect of aECM

The in vivo effect of aECM was investigated. The tumor vessel normalization can be induced by glycolysis inhibition^33^. Therefore, we further studied the tumor vessel normalization caused by aECM treatment. To assess vascular normalization, the three-dimensional structure of vessels in and around the tumor was visualized. Tumors with or without aECM treatment were perfused with silicone rubber radiopaque compound Microfil® (CT angiography agent), and high-resolution µ-CT scanning was performed. The distributions of the polymers in aECM and PBS-treated tumors were compared (Fig. 5d). Within the aECM-treated tumor, the angiographic agent was evenly distributed throughout the tumor. From the large-scale immunofluorescence imaging of tumor vessels, it was also observed that aECM-treated vessels showed a uniform distribution, which were similar to the vessels of normal tissues. To further test whether aECM could normalize the tumor vasculature, CD31 and α-SMA were also stained with immunofluorescent staining. Enhanced fluorescence of α-SMA in CD31^+^ tumor vessels could be seen after aECM treatment, while the fluorescence in the control group was almost invisible (Fig. 5e). To analyze the vessel functionality, dextran-FITC or lectin-FITC was injected into tumor-bearing mice to measure the vessel leakage. Fig. 5f and Supplementary Fig. 11 showed that aECM treatment could increase the perfusion of lectin-FITC in tumor vessels, while the dextran-FITC leakage was reduced by 68%.

Tumor glycolytic markers were also studied to assay the biological effect of aECM. Immunofluorescence staining indicated that GLUT1 was slightly up-regulated (Fig. 5g). Meanwhile, immunofluorescence imaging revealed that aECM-treated tumors had a reduced ability to uptake 2-deoxy-glucose analog (2-NBDG). This result proved that the gelation strategy reduced the ability of the tumors to uptake glucose. Subsequently, to evaluate the influence of aECM on tumor metabolism, glucose concentration and oxygen content were also visualized by using 2-DG 750 (glucose metabolism probe) and HypoxiSense 680 (hypoxia probe). Markedly decreased 2-DG 750 fluorescence intensity was observed in aECM-treated tumors, demonstrating that the glucose uptake was hindered effectively by aECM (Fig. 5h). In contrast, the aECM treatment did not alter the fluorescence intensity of HypoxiSense 680 in tumors. Next, 12-Azidododecanoic acid, an analog of fatty acid, was used to study the effect of aECM in altering lipid transport. Meanwhile, l-azidohomoalaine, an analog of methionine, was used to study the effect of aECM in altering amino acids transport. The intratumoral accumulation of these analogs was analyzed by staining with DBCO-Cy5 through the bioorthogonal reaction between DBCO and N_3_. The ex vivo fluorescence imaging indicated that aECM could suppress the uptake of amino acids, while the lipid transport was almost unaffected (Fig. 5i). These results were in consistence with the in vitro experiments.

### In vivo therapeutic effect of aECM

**Fig. 6:**
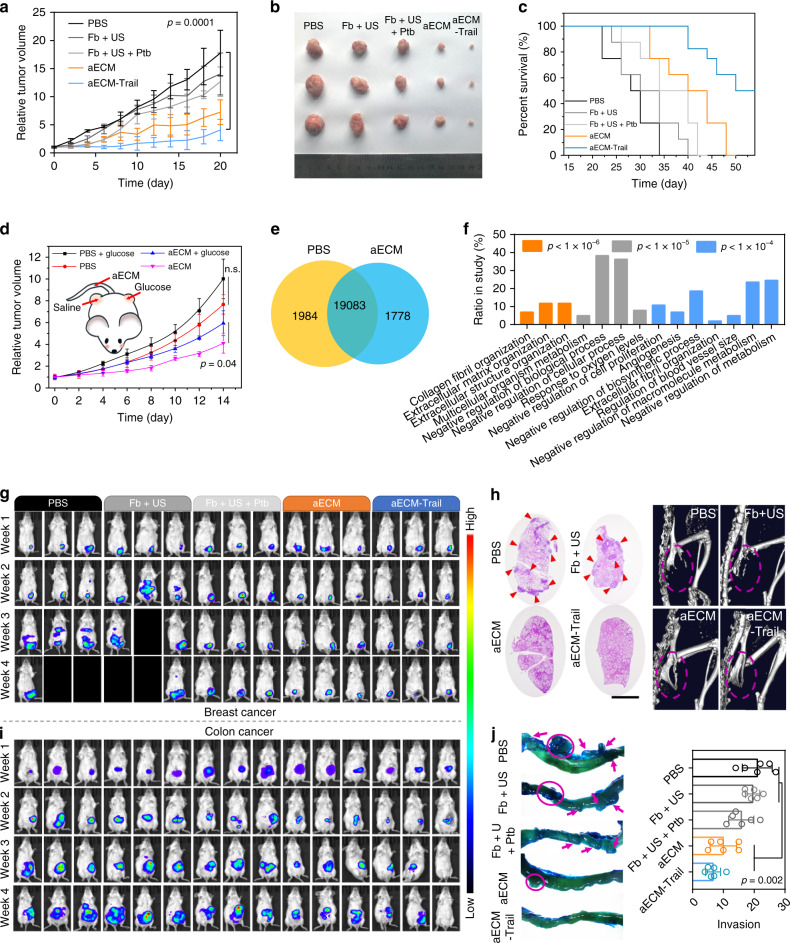
In vivo therapeutic effect of aECM. **a** Tumor volume curve of subcutaneous CT26 tumor-bearing mice after receiving various treatments. The treatment was started (day 0) when the tumor volume reached to 100 mm^3^. Five biological replicates are shown. **b** Typical images of subcutaneous tumors collected after various treatments as indicated. **c** The survival curve of subcutaneous CT26 tumor-bearing mice after receiving various treatments. Eight biological replicates are shown. **d** Intratumoral injection of glucose for rescuing tumor cells from aECM-induced cell death. Four biological replicates are shown. aECM gelation was induced in tumors on both sides of the mice. **e** Venn diagram for the identified genes that between tumors of PBS- or aECM-treated mice (*n* = 3 for each group). **f** Differentially expressed genes were subjected to gene ontology (GO) enrichment analysis. **g** In vivo bioluminescence imaging of orthotopic breast cancer-bearing mice after various treatments. Three representative images of six biological replicates are shown. Black boxes denote dying mice. **h** The anti-metastasis effect of aECM treatment. H&E staining images of lungs from 4T1 tumor-bearing mice after treated with various agents (scale bar: 300 μm). μ-CT images for visualizing the bone metastasis of 4T1 tumor-bearing mice. A representative image of three biological replicates is shown. Arrows denote pulmonary metastasis. Circles mark broken bones. **i** In vivo bioluminescence imaging of orthotopic CT26^luc^ colon cancer-bearing mice after various treatments. Three representative images of six biological replicates are shown. **j** Representative images of intestinal tracts from colon tumor-bearing mice after various treatments. Arrows indicate the invasive tumors. Six biological replicates are shown. Significance between every two groups was calculated by using one-way ANOVA with Tukey post hoc analysis (**a, j**) or (**d**) two-tailed Student’s *t-*test. The BH FDR *p* value (**f**) was used. The mean values and SD are presented.

Then, the in vivo anti-cancer effect of aECM was tested in a subcutaneous CT26 tumor model. The relative tumor volume and the tumor size evaluated on the 20th day exhibited extraordinary tumor regression of aECM (Fig. 6a, b). The tumor growth was suppressed by 56% in aECM-treated group, and 97.2% of suppression was found in aECM-Trail-treated group. After 55 days treatment with aECM-Trail, 50% of the mice were still alive. In a sharp contrast, all the mice in the PBS group died on the 33th day (Fig. 6c). The aECM-treated tumor tissue was observed by TEM. Tumors treated with aECM showed characteristic features of apoptosis (Supplementary Fig. 12). Next, immunofluorescence staining of Ki-67 and TUNEL were used to assess the tumor proliferation. Tumor sections from aECM-Trail-treated mice  showed a notably decreased Ki-67 fluorescence intensity and increased TUNEL fluorescence intensity (Supplementary Fig. 13), indicating the inhibition of tumor proliferation after various treatments. Less frequent metastasis to liver in aECM/aECM-Trail-treated mice was evidenced by H&E staining and anti-luciferase immunofluorescent staining^34^.

To prove that the alteration of mass transports was the dominant mechanism to induce antitumor effects, glucose was intratumorally injected to rescue tumor cells from aECM-induced cell death (Fig. 6d). Before injection of glucose, CT26 cells were subcutaneously injected into both left and right flanks of female BALB/c mice. With the US treatment, the gelation of aECM was initiated in both tumors. In the left tumor, the intratumoral injection of glucose was performed every day to rescue cancer cells from starvation. It was observed that the therapeutic effect of aECM was significantly inhibited after rescuing the tumor starvation, confirming the important role of aECM in inducing starvation. Alterations of mass transports caused by aECM might not be the only reason for its therapeutic effect; nevertheless, it should be a dominated reason. Up-regulation of starvation-associated genes, such as *Ampk* and *Eef2*, also indicated the induction of tumor starvation with aECM treatment (Supplementary Fig. 14)^35^. Transcriptomic analysis was performed on aECM- and PBS-treated tumors. A total of 22,845 genes were identified (Fig. 6e). At a statistical threshold of *p* < 0.05, 115 differentially expressed genes were found (Supplementary Fig. 14). Upregulation genes were enriched in the GO categories of “negative regulation of biosynthetic process”, “negative regulation of macromolecule metabolism”, “negative regulation of metabolism”, and “negative regulation of cell proliferation” (Fig. 6f). This result demonstrated that aECM treatment could systemically affect tumor metabolism and inhibit cell proliferation.

The therapeutic effect of aECM was further evaluated in orthotopic murine models of colon and breast cancers. In an orthotopic breast cancer model, anti-primary tumor and anti-metastasis effects of aECM were investigated. During 4 weeks treatments, more than 50% of the mice died in PBS and Fb + US-treated groups. In contrast, suppressed tumor growth and a higher survival rate were found in aECM and aECM-Trail-treated tumor-bearing mice (Fig. 6g). In PBS- and Fb + US-treated mice, 4T1 cells were found to metastasize from the breast fat pad to distant organs such as lung and liver (Fig. 6h). Bone metastasis is one of the most common complications in patients with breast cancers. μ-CT was also performed to detect the bone metastasis of 4T1 cells. As shown in Fig. 6h, severe bone defects in ischium could be clearly observed in PBS and Fb + US-treated mice, indicating the presence of bone metastasis. None of the mice treated with aECM or aECM-Trail showed any sign of bone defects.

The luciferase expressing CT26 cells were implanted in the cecum of BALB/c mice, and the tumor growth was monitored with the bioluminescence imaging. It was found that mice received aECM-Trail gelation displayed the weakest bioluminescence signal (Fig. 6g). Meanwhile, aECM treatment also substantially inhibited the tumor growth. Furthermore, aECM or aECM-Trail treatment markedly reduced the spread of invasion. After 4 weeks treatments, intestinal tissues of mice were collected and stained with methylene blue. Vast majority of invasive nodes were observed in colons of control mice (Fig. 6j). Images of Ki-67 staining in Supplementary Fig. 15 also revealed that aECM treatment inhibited the cell proliferation in CT26 tumors.

Furthermore, the efficacy of aECM was evaluated on the clinically relevant spontaneous tumor model. C57BL/6J-*Apc*^Min/+^, a spontaneous model for intestinal tumorigenesis was used to test the anti-cancer capacity of aECM. After a 4-week treatment, mice were sacrificed. Their intestinal tissues were collected and stained with methylene blue. The treatment of aECM suppressed the development of colon cancer (Supplementary Fig. 16). From microscopic examination, as compared with the PBS groups, aECM and aECM-Trail treatments suppressed 53% and 59% of adenomas formation, respectively. This result was also confirmed by the H&E staining.

### In vivo combination therapy

**Fig. 7:**
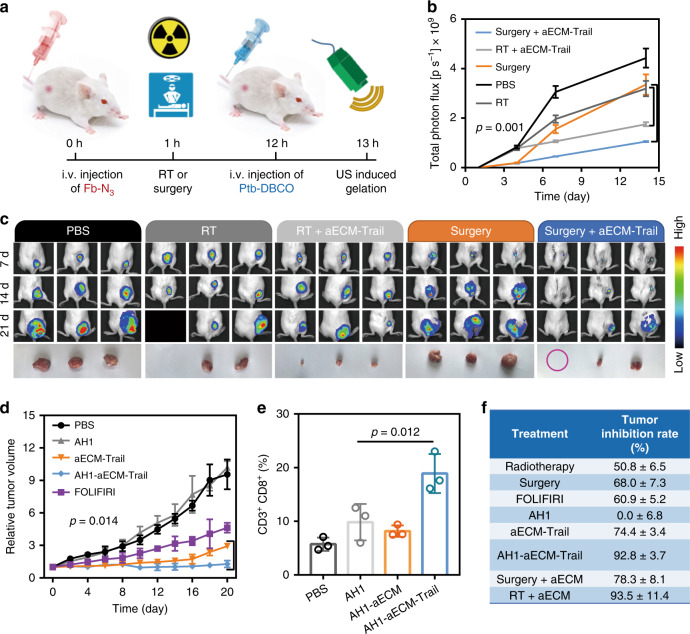
Synergy of aECM with clinical operations. **a** Schematic diagram for the mechanism of surgery and RT (5 Gy) induced aECM gelation. **b** The anti-cancer effect of RT, aECM +  RT, surgery and surgery + aECM towards subcutaneous CT26^luc^ tumor-bearing mice. Bioluminescence intensity of CT26^luc^ tumors was recorded every week. Three biological replicates are shown. **c** In vivo bioluminescence imaging of subcutaneous CT26^luc^ tumor-bearing mice after treatment with aECM gelation and clinical therapies. Mice tumors were collected after a 3-week treatment. Three representative images of five biological replicates are shown. Black boxes denote dying mice. The purple circle represents the invisible tumor. **d** Synergy of aECM with immunotherapy in subcutaneous CT26 ^luc^ tumor-bearing mice Five biological replicates are shown. AH1-aECM, aECM and AH1 peptide (10 mg kg^−1^) and FOLFIRI chemotherapy (30 mg kg^−1^ 5-FU, 90 mg kg^−1^ leucovorin, 16 mg kg^−1^ IRT, i.v.) were given. **e** Quantification of flow cytometric analysis of CD8^+^ CD4^−^ cells gating on CD3^+^ cells. The percentage of CD3^+^ CD8^+^ cells in CD3^+^ lymphocytes was analyzed. Three biological replicates are shown. **f** Comparison of tumor inhibition rates among FOLFIRI chemotherapy, radiotherapy, AH1 immunotherapy (10 mg kg^−1^) and aECM treatment. Significance between every two groups (**b**, **d**, **e**) was calculated by using one-way ANOVA with Tukey post hoc analysis. The mean values and SD are presented.

Besides US treatment, surgery and RT could also make micro-wound and induce the gelation of aECM. Thereafter, combination effects of surgery or RT with aECM against cancer were investigated (Fig. 7a). As shown in Fig. 7b, both surgery and RT exhibited mild therapeutic effects on inhibiting tumor growth. However, the reappearance of tumor at the initial site of the primary tumor was inevitable. In a sharp contrast to that, negligible tumor growth could be found in RT + aECM or surgery + aECM-treated mice. This result was further supported by the in vivo bioluminescence imaging (Fig. 7c). As compared with monotherapies, the combination use of clinical therapies with aECM gelation could achieve more efficient tumor inhibition. From pathological sections and Ki-67 staining, aggressive tumor necrosis and inhibited cell proliferation were found (Supplementary Fig. 17). These results reveal the promising clinical applications of aECM gelation in synergistic tumor therapy.

Given that aECM can specifically accumulate at the tumor site. Therefore, we hypothesized that the conjugation of antigens to aECM might active anti-cancer immune responses. Gp70 is a tumor-specific antigen of murine CT26 tumors. Here, AH1 restricted peptide antigen (SPSYAYHQF) derived from gp70 was conjugated to aECM, and its therapeutic effect was also investigated^36^. It was found that AH1-aECM treatment could reduce the tumor growth in subcutaneous CT26 tumor-bearing mice (Fig. 7d). In agreement with the literature report, the i.v. injection of AH1 peptide did not exhibit significant tumor suppression^37^. The increased cytotoxic T cells (CD3^+^ CD8^+^) in tumors also demonstrated that AH1-aECM could activate the immune response to inhibit tumor growth (Fig. 7e and Supplementary Fig. 18).

Chemotherapy, RT, and surgical resection are common strategies for clinical cancer treatments. Here, a comparison between these therapies with aECM treatment was made in subcutaneous CT26 tumor-bearing mice. As shown in Fig. 7f, some of these therapies (RT and surgical resection) could achieve therapeutic effects similar to those of aECM treatment. However, the aECM treatment still had a list of irreplaceable advantages. First, this non-invasive starvation therapy does not cause side effects in patients. Second, the gelation of aECM can be induced by any clinical operations that accompanied with the induction of blood clotting. aECM is likely to be translated into a clinical combination regimen. Last but not the least, aECM treatment can cause global changes in tumor metabolism, which makes aECM a promising candidate for sensitizing various pharmaceuticals that target abnormal tumor metabolism.

## Discussion

As a proof-of-concept, the intratumoral gelation strategy aimed at trapping solid tumor and cutting off its nutrition supply was developed. By combining the blood clotting response with a bioorthogonal reaction, in vivo gelation with controllable bleeding navigation was successfully induced. The therapeutic efficacy of aECM was evaluated in several mice cancer models. Interestingly, molecules with a strong polarity, low p*K*a, and high molecular weight could be obstructed by aECM. The studies on main metabolic processes including carbohydrate metabolism, amino acid metabolism, and nucleotide metabolism indicate that aECM leads to a nutrition depletion of tumors. Acting as a physical barrier for tumor metastasis, aECM also hinders the spread of cancer cells from entering the circulatory system. Collectively, these two sets of functions improve the therapeutic effect of aECM gelation.

Besides, both Fb and Ptb have been approved by US food and drug administration (FDA) for treating excessive bleeding. Their biological effect and drug safety have been extensively demonstrated in thousands of cases. The investigation of new applications of conditional medications will enhance the translational efficiency of therapies and bring affordable therapeutic regimen for serious illnesses. Our aECM gelation approach based on these two agents is effective and safe in murine models, and promising for the clinical application. However, studies on long-term toxicity and epigenetic effects are necessary for proving the value of these systems. In consequence, detailed dose optimization is still required before the further test in large animal models. Additionally, in rodent models, we demonstrated that the aECM-induced coagulation cascade was effective and safe, whereas, due to the difference of the coagulation system in rodent and primate, this strategy might cause thrombosis in humans. Therefore, research on non-human primate models was still necessary to prove the safety of aECM treatment.

In conclusion, this study developed an enzymatic reaction-based therapy for trapping solid tumors. In principle, aECM gelation could be triggered by any clinical operation with the initiation of blood clotting. In this study, with the combination of US, surgery, or RT, the aECM gelation was successfully induced in various cancer types. This capacity also establishes the benefit of introducing aECM gelation to standard therapeutic regimens for the first-line treatment of the majority of cancer cases. Although such enzymatic reaction-based therapy is now still in its infancy at the present, we believe this strategy will offer a brand new avenue to update current cancer therapies and benefit personalized medicine.

## Methods

### Study design

The objective of this study was to develop chemical modified clotting associated proteins to enable besiege of tumor and cut off its nutrition supply. The gelation of aECM was proved to be induced by clinical operations such as US treatment, surgery, or RT. The in vivo antitumor efficacy was assessed in CT26 and 4T1 tumor models. Sample sizes were determined on the basis of our previous experimental experience. Animals were randomly assigned to different groups. The investigators were not blinded to allocation during experiments and outcome assessment.

### Cell culture

4T1 mammary cancer cells and CT26 cells were cultured in 1640 medium with 5% CO_2_ at 37 °C, MCF-7 cancer cells were cultured in MEM medium with 5% CO_2_ at 37 °C, and HT29 cancer cells were cultured in McCoy’s 5a modified medium with 5% CO_2_ at 37 °C. The 1640 medium, MEM medium, and McCoy’s 5a medium modified medium contain 10% heat-inactivated FBS and 1% antibiotics (penicillin–streptomycin, 100 U mL^−1^).

### Materials

Calcium chloride (CaCl_2_), glucose, spermidine, lactic acid, NaNO_2_, acrylic amide, GSH, fluorescamine, 5,5′-dithiobis-(2-nitrobenzoic acid) (DTNB), thrombin, FITC-dextran, fluorescein, and rhodamine B (RB) were purchased from Aladdin Industrial Corporation. Gadopentetate dimeglumine (Gd-DTPA), sodium diatrizoate, Trail, fibrinogen, and prothrombin complex were purchased from Sigma-Aldrich. Hoechst 33342 and DAPI were purchased from Thermofisher Scientific. TUNEL assay, ATP Assay Kit, Lactate Assay Kit, Live/dead cell stain assay, and 3-(4,5-dimethyl-2-thiazolyl)-2,5-diphenyl-2-H-tetrazolium bromide (MTT) assay were purchased from Beyotime Biotechnology. Cy5-NHS and Cy7-NHS were purchased from AmyJet Scientific Inc. ROS-ID was obtained from Enzo Life Sciences. 2-DG-750 probe and HypoxiSense 680 probe were purchased from PerkinElmer Inc. DBCO-PEG_4_-NHS and N_3_-PEG-NHS were purchased from Ponsure. Micro-fluidic chip was purchased from Suzhou WenHao Microfluidic Technology Co., Ltd. Recombinant Trail protein was purchased from Bioxcell.

### Antibody

The primary antibodies for immonofluorescence were anti-Ki-67 rabbit monoclonal antibody (GB11030, dilution: 1:200) from Servicebio, anti-alpha smooth muscle actin antibody [E184] (ab32575, dilution: 1:200), anti-CD31 antibody [P2B1] (ab24590, dilution: 1:200), and anti-glucose transporter GLUT1 antibody [EPR3915] (ab115730, dilution: 1:200) from Abcam. Fibrinogen alpha chain polyclonal antibody (20645-1-AP, dilution: 1:100) was purchased from Proteintech. FITC anti-mouse CD3 antibody [17A2] (100203, 5 μg mL^−1^), PE anti-mouse CD4 antibody [GK1.5] (100407, 2.5 μg mL^−1^), and APC anti-mouse CD8a antibody [53-6.71] (100711, 2.5 μg mL^−1^) were purchased from Biolegend.

### Animal

Experimental protocols were approved by the Institutional Animal Care and Use Committee (IACUC) of the Animal Experiment Center of Wuhan University (Wuhan, China). All animal experimental procedures were performed in accordance with the Regulations for the Administration of Affairs Concerning Experimental Animals approved by the State Council of People’s Republic of China. Animals were housed in groups of 4–6 mice per individually ventilated cage in a 12 h light dark cycle (6:30–18:30 light; 18:30–6:30 dark), with constant room temperature (21 ± 1 °C) and relative humidity (40–60%). Animals had access to food and water ad libitum.

### The formation of aECM and SEM sample preparation

In vitro aECM was referred to the fibrinogen proteolytically activated by thrombin to form gel-like clots. Briefly, aECM was prepared by adding thrombin (1 mg mL^−1^, 50 μL) to fibrinogen (3 mg mL^−1^, 0.5 mL) in 24-well plates and standing for 5 min to activate the fibrinogen adequately for gel-like clots formation.

The prepared clots were separated followed by soaking in 2.5% glutaraldehyde solution for 1 h. Clots were then washed with DI water for five times (5 min each time) and dehydrated in turn with 25, 50, 75, 100, and 100% ethanol solutions (10 min for each concentration). Next, samples were placed in a mixture of hexamethyldisilazane and ethanol (1:3 volume ratio) for 15 min and subsequently dipped into the pure hexamethyldisilazane for 15 min. Samples were dried overnight in the air.

### The formation of aECM in vivo

The in vivo gelation of aECM was proteolytically activated by micro-wound to amply clotting cascade in tumor regions. In subcutaneous models, the formation of aECM was performed as follows. First, Fb-N_3_ was injected intravenously (5 mg mL^−1^, 100 μL) to the mice. For the ultrasound group, 1 h later, a US treatment (600 W, 5 s) was carried out in the tumor position to trigger the gelation of fibrinogen. For the RT group, a 5-Gy dose of superficial 6 MV photon irradiation was given within 5 min. For the surgery group, a horizontal incision of 5 mm in length was made by using an endotherm knife. The tumor visible to the naked eye was completely removed, and the incision site was closed with one drop of Vetbond tissue adhesive. 12 h after these treatments; Ptb-DBCO (2 mg mL^−1^, 100 μL) was injected through tail vein. A second US treatment (600 W, 5 s) was performed to induce the clotting. For orthotopic and spontaneous tumor models, the US treatment with longer time was used. In each US treatment, the ultrasound probe was performed for three cycles of 5 s at an interval of 30 s (600 W). The accumulation of Fb-N_3_ was measured with in vivo fluorescence imaging and fibrinogen ELISA kit (Genetex). The reaction between Fb-N_3_ and Ptb-DBCO was measured with in vivo fluorescence imaging. The successful induction of clotting response was measured with thrombin activity fluorometric assay kit (Biovision).

### Diffusion of metabolites through aECM

The diffusion assay was performed on a Transwell insert. The aECM-coated Transwell insert was prepared by adding thrombin (1 mg mL^−1^, 50 μL) to fibrinogen (3 mg mL^−1^, 0.5 mL) in 24-well Transwell insert (8 μm) and standing for 5 min to activate the fibrinogen adequately for gel-like clots formation. The collagen-coated Transwell insert was set as a control. Two hundred microliters of the 1640 medium containing rat tail type I collagen (100 mg mL^−1^) was gelled in the upper chamber of Transwell inserts and incubated at 37 °C for 6 h. Then, the gel was washed thrice with PBS and dried at 37 °C. The concentration gradient between upper and lower chambers is the driving force for diffusion. For the preparation of oxygen-condensed solution, 50 mL of PBS was placed into a round bottom flask, and the solution was saturated with oxygen at room temperature by bubbling with oxygen gas for 15 min. The solution was used as soon as possible after preparation. An oxygen dissolving meter was used to measure the oxygen concentration, and the oxygen concentration of as-prepared solution was around 8.4 mg L^−1^. The deoxygenated solution (O_2_: ~1 mg L^−1^) was prepared by purging high-purity N_2_ for at least 2 h. In order to establish an oxygen gradient, the deoxygenated solution was added to the up-chamber, and the oxygen-condensed solution was added to the down-chamber. These solutions were used immediately after the preparation. Firstly, the diffusion effect of five typical metabolic molecules (spermidine, lactate, glucose, GSH, and O_2_) was studied by fluorescamine assay, lactate assay kit, glucometer, DTNB assay, and dissolved oxygen meter, respectively. Briefly, aECM was prepared to cover on the membrane of upper chamber. Then, the solution containing five typical metabolic molecules was placed to the lower chamber and DI water (2 mL) was added in the upper chamber, leading to a sharp concentration gradient between two chambers. The concentration of metabolic molecules in upper chamber was tested every 5 min.

The diffusion of serum metabolites was further investigated. The BALB/c mice were anesthetized with isoflurane. Five hundred microliters of whole blood was collected from mice heart and placed in a coagulation tube for 1 h. Serum was isolated by centrifugation at 3000 *g* for 10 min and was stored at −80 °C. The mice serum (protein content of 10 mg mL^−1^; 2 mL) was placed in lower chamber of the Transwell insert and DI water (2 mL) was added to the upper chamber. Five hundred microliters of solution in upper chamber were collected every 30 min and measured with a GC/MS.

### In vivo therapeutic effect

Female BALB/c mice (5–6 weeks) subcutaneously xenografted with CT26 tumor were used to evaluate the in vivo therapeutic effect of aECM. CT26 cells (1 × 10^6^) were implanted on the right flank of BALB/c mice. The mice were randomly divided into five groups (*n* = 5 for each group). When the tumor volume reached to 100 mm^3^, the mice were respectively treated with PBS, Fb-N_3_ + US, Fb-N_3_ + US + Ptb-DBCO, aECM (Fb-N_3_ + US + Ptb-DBCO + US), and aECM-Trail (Trail-Fb-N_3_ + US + Ptb-DBCO + US). The treatment was given every 3 days for a total of three times (day 1, day 4 and day 7). For the treatment of aECM-Trail or AH1-aECM, a similar dose of Trail-Fb-N_3_ or AH1-aECM, instead of Fb-N_3,_ was used. AH1 restricted peptide antigen (SPSYAYHQF) derived from gp70 was given (i.v.) at a dose of 10 mg kg^−1^. In order to compare the therapeutic effects of aECM with chemotherapy, tumor-bearing mice receiving intravenous FOLIFIRI (30 mg kg^−1^ of 5-fluorouracil, 90 mg kg^−1^ of leucovorin, 16 mg kg^−1^ of irinotecan) treatment, a standard chemotherapy regimen for advanced colon cancer, was set as a control (*n* = 5). The chemotherapy was given once a week. Tumor volumes and body weight were measured every 2 days during the treatment.

The therapeutic effect of aECM was further evaluated in orthotopic murine models of colon cancer and breast cancer. First, 1 × 10^6^ luciferase-expressing CT26 cells (CT26^luc^) dispersed in ~40 µL of PBS were injected directly into the cecum of BALB/c mice to establish an orthotopic colon cancer mouse model. The luciferase-expressing 4T1 cells (4T1^luc^, 5 × 10^6^ cells) were injected in their right side of mammary fat pad to establish an orthotopic breast cancer model. The mice in two orthotopic murine models were randomly divided into five groups (*n* = 6 for each group). Three days after the tumor inoculation, the mice in both models were treated with PBS, Fb-N_3_ + US, Fb-N_3_ + US + Ptb-DBCO, aECM, and aECM-Trail, respectively. Various treatments were given every three days for three times. At 7th, 14th, and 21st days post treatment, the tumor volumes were measured with the intratumoral bioluminescence intensity in IVIS Spectrum (PerkinElmer). Intraperitoneal injection of d-luciferin solution (30 mg mL^−1^, 100 μL) was performed 20 min before imaging. Over time, 4T1 cells in the orthotopic breast tumor would spontaneously metastasize to bone and lung. In the orthotopic colon cancer model, CT26 cells would gradually invade surrounding tissues. The bone metastasis of breast cancer was visualized with the Quantum GX microCT Imaging System (PerkinElmer). The lung metastasis of breast cancer was observed from H&E images. Intestinal tissues from orthotopic CT26 tumor-bearing mice were stained with methylene blue and observed with a stereomicroscope.

### Surgery/RT induced gelation for cancer treatment

The combinations of aECM with clinical therapy including surgery and RT were studied. The CT26^luc^ cells (1 × 10^6^) were implanted on the right flank of BALB/c mice. The mice were randomly divided into five groups at 3 days post-tumor inoculation (*n* = 5 for each group). When the tumor volume reached 200 mm^3^, the mice were treated with PBS, RT, surgery, RT + aECM, and surgery + aECM, respectively. First, Fb-N_3_ was injected intravenously (5 mg mL^−1^, 100 μL) to the mice. For the radiotherapy group, a 5-Gy dose of superficial 6 MV photon irradiation was given within 5 min. For the surgery group, a horizontal incision of 5 mm in length was made using small surgical scissors. The tumor visible to the naked eye was completely removed, and the incision site was closed with one drop of Vetbond tissue adhesive. Twelve hours after these treatments, Ptb-DBCO (2 mg mL^−1^, 100 μL) was injected through tail vein. A second US treatment (600 W, 5 s) was performed to induce the clotting. The treatment was given every 3 days for a total of three times. The tumor volume and body weight were measured every 2 days during the process of the treatment. In vivo fluorescence imaging was also carried out to measure the tumor growth.

### The combination of aECM with immunotherapy

BALB/c mice (5–6 weeks) subcutaneously xenograft with CT26 cells (1 × 10^6^) were used. AH1-A5 peptide, a truncated peptide of CT26 gp90 antigen, was combined with aECM for synergistic treatment. When the tumor volume reached 100 mm^3^, the mice were treated with PBS, AH1-A5 (20 μg per mice), aECM, and AH1-A5 (20 μg per mice) + aECM (*n* = 5 for each group), respectively. During the treatment, the tumor volumes were measured every 2 days. The treatment was given every 3 days for a total of three times. Twenty days later, mice were sacrificed and their tumors were collected. The number of CD3+CD8+ T cells and CD4+CD4+ T cells in the tumor was studied by flow cytometry.

### The anti-cancer effect of aECM in *Apc*^*Min+/*^^−^ mice

The anti-cancer effect of aECM was also evaluated in C57BL/6J-*Apc*^*Min/+*^ mice with spontaneous colon cancer. Eight-week-old C57BL/6J-*Apc*^*Min/+*^ mice were randomly divided into four groups with five mice per group. Fb-N_3_ was injected intravenously (5 mg mL^−1^, 100 μL) to the mice. One hour later, the US treatment was carried out in the tumor position to trigger the gelation of fibrinogen. Twelve hours after these treatments, Ptb-DBCO (2 mg mL^−1^, 100 μL) was injected through tail vein. A second US treatment was performed to induce the clotting. In each US treatment, the ultrasound probe (600 W) was applied for three cycles of 5 s at an interval of 30 s. The treatment was performed once a week. After 4 weeks of therapy, mice were sacrificed. Their intestinal samples were collected for observation and histological analysis.

### Reporting summary

Further information on experimental and research design is available in the Nature Research Reporting Summary linked to this article.

## Supplementary information

Supplementary Information

Reporting Summary

## Data Availability

The source data underlying Figs. 1e, f, 2a–e, h, 3a–b, 4a, h, 5c, f, 6a, c, d, f, j, 7b, d, e and Supplementary Figs. 1, 2, 6–11, and 13–17 are provided as a Source Data file. All the relevant data are available from the authors upon reasonable request. The transcriptomic data are available at NCBI under Project PRJNA629444. A reporting summary for this article is available as a Supplementary Information file. Source data are provided with this paper.

## Source data

Source Data
