# Supplementary material for: Controllable gelation of artificial extracellular matrix for altering mass transport and improving cancer therapies
Source: Nat Commun. 2020 Sep 30;11:4907. doi: 10.1038/s41467-020-18493-7 (PMC7527557; doi:10.1038/s41467-020-18493-7)
Supplement: Supplementary file 1 — Supplementary Information [file 41467_2020_18493_MOESM1_ESM.pdf]

Supplementary Information of

Controllable gelation of artificial extracellular matrix for altering mass transport and improving cancer therapies

Zheng *et al.*

Characterization. Ultraviolet & visible diffuse reflectance spectroscopy (UV-vis DRS) was carried out on a Shimadzu UV-3600 UV-vis spectrophotometer. Scanning electron microscopy (SEM) images were obtained with a scanning electron microscope (Zesis Sigma). Small animal fluorescence imaging was performed with a living image IVIS® spectrum (Perkin-Elmer). Confocal laser scanning microscope (CLSM) images were obtained on a C1-Si (Nikon) confocal laser scanning microscope. Intravital fluorescence images were captured on a Nikon fluorescent microscope equipped with a digital camera (TE2000-U, Nikon). μ-CT images were obtained from Quantum GX micro-CT imaging system (Perkin-Elmer). MRI images were obtained on a 7.0 T MRI equipment (BioSpec 70/20USR). The radiotherapy was performed within Unique™ radiation therapy system (Varian).

Preparation of azido-modified fibrinogen (Fb-N_3). The azidation of fibrinogen was conducted with N_3-PEG_4-NHS in PBS buffer at pH 7.4. Briefly, 20 mg of fibrinogen and 2 mg of N_3-PEG_4-NHS were dissolved in 5 mL PBS (pH=7.4) and reacted for 4 h. Fb-N_3 containing fractions was then collected and purified with ultrafiltration membranes (MwCO 10 kDa) to remove unbound N_3-PEG_4-NHS.

Preparation of azodibenzocyclooctyne grafted prothrombin (Ptb-DBCO). Ptb-DBCO was prepared by conjugating DBCO-PEG_{2000}-NHS on the prothrombin in PBS buffer at pH 7.4. Briefly, 5 mg of prothrombin and 1 mg of DBCO-PEG_{2000}-NHS were dissolved in 1 mL PBS (pH=7.4) and reacted for 4 h. Ptb-DBCO containing fractions was then collected and purified with ultrafiltration membranes (MwCO 10 kDa) to remove unbound DBCO-PEG_{2000}-NHS.

Conjugation of Trail to Fb-N_3. A method of coupling Trail to Fb-N_3 was managed by using EDC/NHS chemistry. To conjugate Trail with Fb-N_3, 6 mg of Fb-N_3 was suspended in 3 mL PBS buffer. 0.5 mL of PBS containing 0.48 mg of 1-(3-dimethylaminopropyl)-3-ethylcarbodiimide hydrochloride (EDC.HCl, 2.5 mM) and 0.29 mg of N-hydroxysuccinimide (NHS, 2.5 mM) were added to the Fb-N_3 solution and stirred at room temperature for 4 h. The product was collected via dialysis in phosphate buffered saline (PBS) to remove unreacted EDC.HCl and NHS. Trail was then added and the mixture was stirring at room temperature for another 4 h. The resulting Trail-Fb-N_3 was purified with ultrafiltration membranes (MwCO 50 kDa) to remove unbound Trail.

Conjugation of diatrizoic acid (DA) to Fb-N_3. To conjugate DA to Fb-N_3, 6 mg of DA was suspended in 4 mL PBS buffer. Equal amounts of EDC and NHS were added to the DA suspension and stirred at room temperature for 2 h to activate DA. Fb-N_3 was then added and the mixture was reacted for another 1.5 h. The resulting DA-Fb-N_3 was collected and purified with ultrafiltration membranes (MwCO 10 kDa).

The fluorescence labeling of proteins. Rhodamine B isothiocyanate labeled Fb-N_3 was managed by using the reaction between rhodamine B isothiocyanate and Fb-N_3. In brief, 10 mg of Fb-N_3 was suspended in 5 mL PBS buffer. 0.5 mg of the rhodamine B isothiocyanate was added to the Fb-N_3 solution and stirred for 4 h. Resulting rhodamine B labeled Fb-N_3 was collected and purified with ultrafiltration membranes (MwCO 10 kDa) to remove unbound rhodamine B isothiocyanate.

The labeling of Fb-N_3 and Ptb-DBCO was conducted respectively with Cy7-NHS

and Cy5-NHS in PBS buffer at pH 7.4. 10 mg of Fb-N_3 or Ptb-DBCO was suspended in 5 mL PBS buffer. 0.5 mg of dyes were added to the Fb-N_3 solution and stirred for 4 h. The products were then collected and purified with ultrafiltration membranes (MwCO 10 kDa) to remove unbound dyes.

Cell migration assay. In the cell migration experiment, CT26 cells were seeded in the 24-well plates (5×10^5 cells per well) and cultured for 24 h. The medium was removed and the cells were washed twice with Versene followed by adding 1 mL of Versene (containing 0.05% trypsin) and keeping for 10 to 15 min at 37 °C. 3 mL of DMEM (10% FBS) was added followed by centrifugation at 150-200 g for 5 min. The medium was removed and the cells were resuspended in 1.5 mL of DMEM for later use. The prepared CT26 cells were then added into the top chambers of Transwell. Then, aECM was prepared by adding thrombin (1 mg mL^{-1}, 10 μL) and Fb (3 mg mL^{-1}, 0.1 mL) into the cells for forming gel-like clots. The lower chambers were added with 500 μL of 1640 medium as a chemoattractant. After incubation with aECM or PBS for 48 h, the number of cells migrated to the lower chambers was counting under a light microscope at 40 × magnification (n = 5).

Cell invasion assay. 200 μL of the 1640 medium containing rat tail type I collagen (100 mg mL^{-1}) was gelled in the upper chamber of Transwell insert and incubated at 37 °C for 6 h. The gels were washed thrice with PBS and dried at 37 °C. The prepared CT26 cells were then added into the top chambers of collagen-coated Transwell insert. Then, aECM was prepared by adding thrombin (1 mg mL^{-1}, 10 μL) and Fb (3 mg mL^{-1}, 0.1 mL) in to the cells for forming gel-like clots. The lower chambers were added with 500

μL of serum-containing 1640 medium as a chemoattractant. After incubation with aECM or culture medium for 48 h, the number of cells migrated to the lower chambers was counted under a light microscope at 40 × magnification (n = 5).

Tracking experiments for cancer cell motility. To achieve long-term observation of cell migration, microscope stage-top incubator system was used. Tracking experiments for cancer cell motility were performed over 11 h. During the observation, 4T1 cells were cultured in a 5% CO_2 atmosphere at a constant temperature of 37 °C. The movement routes of CT26 cells within 11 h were analyzed with an Image J plug-in, MTrack2. Both speed and mean squared displacement values were quantitatively analyzed from 100 cells from 4 repeated experiments by using Image J plug-in, MTrack2.

The preparation of multicellular tumor spheroids (MTS). 1% (w/v) of agarose gel was covered on the 96-well plates to prevent the cell adhesion. Then, cancer cells (4T1, CT26, MCF-7 or HT29 cells) were seeded into wells (about 1000 cells per well) and agitated for 5 min followed by incubation at 37 °C for 7 days. The uniform and compact MTS were used for the further studies.

The disassembly of MTS. The disassembly of MTS was carried out by using a shake cultivation. MTS in 96-well plates was treated with aECM or culture medium. After being washed with PBS for three times, MTS was shaken on a shake cultivation at 300 rpm. The disassembly of MTS was observed by fluorescent microscope every 2.5 min.

Cell viability study. The cell viability was firstly studied in 2D cell culture system. CT26, 4T1, MCF-7 and HT29 cells were respectively seeded in 96-well plates (5×10^3

cells per well). After 24 h of growth, the cells were treated with aECM for another 24 h. The cell viability was also studied in 3D MTS model. The prepared MTS was treated with aECM for 24 h. After being washed with PBS for three times, the cells were added with 10 μL of CCK-8 solution and cultured for 2 h. The UV-vis absorptions at 450 nm (test wavelength) and 690 nm (reference wavelength) of cells were measured using a microplate reader. Cell viability (%) was calculated, and data was presented as mean ± standard deviation (SD) in triplicate.

***In vitro* metabolic study.** CT26, 4T1, MCF-7 and HT29 cells were respectively seeded in 96-well plates (5×10^3 cells per well). After 24 h of growth, the cells were treated with aECM for another 24 h. For the 3D MTS model, 1% (w/v) of agarose gel was covered on the 96-well plate to prevent cell adhesion. Then, cancer cells (4T1, CT26, MCF-7 or HT29 cells) were seeded into wells (about 1000 cells per well) and agitated for 5 min followed by incubation at 37 °C for 7 days. MTS were treated with aECM for another 24 h. The extracellular O_2 level was measured with MitoXpress xtra oxygen consumption assay (Luxcel Biosciences). The extracellular lactate accumulation was measured with MitoXpress & pH-xtra consumption assay (Luxcel Biosciences). The intracellular ATP level was measured with ATP assay kit (Beyondtime). The intracellular O_2 level was measured with ROS-ID (Enzo life sciences).

Enzymatic degradation of aECM *in vitro*. The degradation of aECM by MMP-2 *in vitro* was studied using FITC-labeled aECM (FITC-aECM). Briefly, the FITC-aECM was prepared by adding thrombin (1 mg mL^{-1}, 50 μL) to FITC-labeled fibrinogen (3 mg mL^{-1}, 0.5 mL) in 24-well plates and standing for 5 min at 37 °C to activate the

fibrinogen adequately for gel-like clots formation. Then, the clots were separated and washed by PBS several times until almost all the free FITC-labeled fibrinogen was removed. The prepared FITC-aECM was incubated in PBS supplemented with or without MMP-2 (1.5 μg mL^{-1}) at 37 ºC. MMP-2 was added every 2 days and the fluorescence of the supernatant was recorded every day. The hydrolysis rates were calculated.

Enzymatic hydrolysis of aECM *in vivo*. The BALB/c mice with CT26 cell (1×10^6 cells per mouse) tumors inoculated on their right flank were used for *in vivo* aECM enzymatic hydrolysis study. When the tumor volume reached to ~100 mm^3, the *in vivo* gelation was induced. Cy7-Fb-N_3 was injected intravenously (5 mg mL^{-1}, 100 μL) to the mice. 1 h later, an ultrasonic treatment (600 W, 5 s) was carried out in the tumor position to trigger the gelation of fibrinogen. 12 h after these treatments, Ptb-DBCO (2 mg mL^{-1}, 100 μL) was injected through tail vein. A second ultrasonic treatment (600 W, 5 s) was performed to induce the clotting. For the control group, the pan MMPs inhibitor, Batimastat, was intraperitoneal injected at a dose of 30 mg kg^{-1}. Live animal fluorescent imaging (IVIS) was used to track intratumoral degradation of Cy7 labeled aECM at 1st, 5th and 10th days.

Metabonomics and transcriptomics analysis. Metabonomics analysis was carried out according to the previous work of our group. The BALB/c mice with CT26 cell (1×10^6 cells per mouse) tumors and 4T1 cell (1×10^6 cells per mouse) tumors inoculated on their right flank were used for metabonomics analysis. When the tumor volume reached to ~100 mm^3, aECM gelation was induced. CT26 tumors and 4T1 tumors were

collected 3 days after the gelation and stored at −80 °C for later use.

For the metabonomics analysis, tumor samples were then mixed with 1 mL extractant (acetonitrile:isopropanol:water = 3:3:2) followed by ultrasonic for 1 min under ice bath. Subsequently, the mixture was centrifuged (1300 g, 5 min) and the supernate (800 μL) was mixed with Myristic acid-*d27* (3 mg mL^{-1}, 20 μL). The mixture was then blow-dried with nitrogen gas followed by adding methoxyamine hydrochloride / pyridine (40 mg mL^{-1}, 20 μL) for 90 min at room temperature. The mixture was mixed with N-methyl-N-(trimethylsilyl) trifluoroacetamide (90 μL) and 1% trimethylchlorosilane (MSTFA + 1% TMCS) and then co-incubated at 37 °C for 30 min. After centrifugation, the supernatant (80 μL) was collected for subsequent GC-MS analysis by using an Agilent 7890 GC-Agilent 5975c inert MSD with a triple-axis detector. The comparison of GC separation was analyzed by a commercial GC capillary column (Agilent ZORBAX DB5-MS, 30 mm length × 0.25 mm i.d. × 0.25 μm) (Agilent Technology).

Vascular normalization. For obtaining the three-dimensional structure of the vessels in and around the tumor, μ-CT scanning was performed on aECM treated tumors (n = 3 for each groups). Mice were sacrificed and perfused intracardiacally with silicone rubber radiopaque compound Microfil (FlowTech, Carver, MA), which polymerized in blood vessels within 20 minutes. After Microfil perfusion, the tumor was excised and preserved in 4% formalin. The blood vessels in the tumor were scanned with μ-CT. The three-dimensional image of the tumor blood vessels was reconstructed by the software that comes with the instrument.

The vascular leakage and perfusion were studied by intravenous injection of FITC-dextran and FITC-lectin. Extravascular diffusion was assessed by intravenous injection with FITC-dextran (40 kDa, 0.25 mg per mouse). 10 min after the injection, mice tumors were collected. Cryosections with a thickness of 20 μm were obtained. The blood vessels were stained with anti-CD31 antibody (red fluorescence). The FITC-dextran was calculated as the ratio of green fluorescence to red fluorescence.

The vascular perfusion was assessed by intravenous injection with FITC-lectin (10mg kg^{-1}). 10 min after the injection, mice tumors were collected. Cryosections with a thickness of 20 μm were obtained. The blood vessels were stained with stained anti-CD31 antibody (red fluorescence). The overlap ratio between lectin+ area and CD31+ area was calculated.

***In vivo* fluorescence imaging.** In order to prove the *in vivo* gelation of aECM, intravital fluorescence microscopy was performed to visualize this process. To establish the ear tumor model, 50 μL 4T1 cell suspension (containing 1×10^6 cells) was injected in the peripheral ear. 7 day after the injection, the mouse was anesthetized by using isoflurane followed by injection with 50 μL FITC-labeled dextran (FITC-dextran; 5%, 150 kDa). The ear of the mouse was settled on the microslide by coating a piece of coverslip on the microslide to clamp the ear. The animal was then moved to the desk of the fluorescence microscope. The fluorescence microscope was adjusted for FITC-dextran visualization. And then the mouse was injected with RB-labeled fibrinogen followed by ultrasound. The vessel was observed at intervals. Adequate depth of anesthesia should be ensured during all the process.

The BALB/c mice with CT26 (1×10^6 cells per mouse) tumors inoculated on their right flank were also used to study the *in vivo* fluorescence imaging**.** When the tumor volume reached to ~200 mm^3, mice were intravenously injected with Cy7-Fb-N_3 and Cy5-Ptb-DBCO followed by treatment with ultrasonic in the tumors. Then, the fluorescence imaging of mice was performed by an IVIS Spectrum (PerkinElmer) at different time points.

Additional glucose for impairing the therapeutic effect of aECM. CT26 cells suspended in PBS were subcutaneously injected (1×10^6) into both left and right flanks of female BALB/c mouse (5-6 weeks). With the US treatment, the gelation of aECM was initiated in both mice tumors. In the left tumor, the intratumoral injection of glucose solution with a dose of 1g kg^{-1} was performed every day. Meanwhile, the same volume of saline was intratumoral injected into the right tumor. The tumor volume was recorded every two days.

Tissue clearing and 3D tumor fluorescence imaging. Tissue clearing was performed on CLARITY technique. CT26 tumor-bearing mice were sacrificed after aECM treatment for 24 h followed by perfusion with 0.5% $NaNO_2$ and 10 U mL^{-1} heparin contained ice-cold PBS. And then the tumors of mice were collected and mixed with 4% paraformaldehyde at 37 °C for 2 h. The tumors were then soaked in acrylamide monomer (4%) and 2,2'-azobis [2-(imidazolin-2-yl)propane] dihydrochloride thermoinitiator (0.25%) contained ice-cold PBS at 4 °C for 1 day to ensure enough penetration of the monomer and initiator. Then, the polyacrylamide hydrogel was kept in 37 °C water with N_2 protection to initiate the polymerization. After 3 h of

polymerization, the system was then washed with PBS contained 0.01% NaN_3 and 10% SDS by keeping in 37 ºC water for 4 days until became optically transparent hydrogel. The CLARITY method could only remove lipid molecules without removing biomacromolecules such as proteins or nucleic acids among tissues. Thus, further immunofluorescence staining could be performed. The transparent hydrogel obtained by CLARITY method was then stained with DAPI for 15 min and washed with PBS for several times. The 3D tumor fluorescence imaging was observed on a C1-Si (Nikon) confocal laser scanning microscope.

***In vivo* CT imaging of fibrinogen.** The BALB/c mice with CT26 cell (1×10^6 cells per mouse) tumors inoculated on their right flank were used for *in vivo* CT imaging (n = 3 for each groups). The *in vivo* CT imaging was performed on a Quantum FX micro-CT imaging system (Perkin-Elmer) by using diatrizoic acid conjugated Fb-N_3. When the CT26 tumor volume reached to ~200 mm^3, the *in vivo* gelation of aECM was induced. A similar dose of DA-Fb-N_3, instead of Fb-N_3, was used. The CT imaging was recorded before and after treatment with ultrasonic.

MRI imaging for the permeability of the tumor. The BALB/c mice with CT26 cell (1×10^6 cells per mouse) tumors inoculated on their both side of flank were used for *in vivo* permeability study. The *in vivo* MRI imaging was performed on a 7.0 T clinical MRI instrument and the T1-weighted MRI scanning was performed on the bilateral tumor-bearing mice (n = 3 for each groups). Briefly, when the tumor volume reached to ~100 mm^3, the right tumor of each mouse was treated with aECM through subcutaneous injection and the left one was injected subcutaneously with

equivoluminal PBS as the control. After 12 h post-administration, the contrast agent gadopentetate dimeglumine (0.1 M, 150 μL) was injected intravenously to the mice. And T1-weighted MRI scans were performed consecutively at 10 min, 40 min and 2 h after the injection of gadopentetate dimeglumine (0.1 M, 150 μL).

Hemolysis assay. Blood samples were obtained from heart of mice by using blood collection tube containing Na-heparin as an anti-coagulant. Then, 100 μL Blood samples were respectively added into 900 μL of PBS (as negative control), deionized water (as positive control), Fb and aECM. After co-incubation in shaking incubator for 4 h at 37 ºC, the mixtures were centrifuged (300 g, 5 min) to remove unbroken red blood cell and the clots. The supernates were then added into a 96-well plate followed by measuring their absorbance at 540 nm by using a microplate reader. The data are presented as mean ± standard deviation (SD) in triplicate.

Blood routine examination and blood biochemistry analysis. To evaluate the effects in physiology caused by aECM, the blood biochemical indexes and blood routine indexes were tested. Female BALB/c mice were divided into 3 groups (n = 3 for each groups) and treated respectively with PBS, aECM and aECM-Trail *via i.v.* administration. After 3 days of post-administration, blood samples were collected from heart (100 μL each mouse). The blood routine examination was carried out by Auto Hematology Analyzer (MC-6200VET), and blood biochemistry analysis was performed by biochemical auto analyzer (MNCHIP, Tianjin, China).

Flow cytometry. Before staining, the cells were incubated with mouse anti-CD16/32 mAb (Bioleglend, CAT.NO. 101329, Clone: 93, 10 μg mL^{-1}) to block non-specific Fc

binding. Then, cells were stained with FITC Anti-mouse CD3 Antibody (Biolegend, CAT.NO. 100203, Clone: 17A2, 5 μg mL^{-1}), PE Anti-mouse CD4 Antibody (Biolegend, CAT.NO. 100407, Clone: GK1.5, 2.5 μg mL^{-1}), and APC Anti-mouse CD8a Antibody (Biolegend, CAT.NO. 100711, Clone: 53-6.7, 2.5 μg mL^{-1}). Unstained cells and cells stained with CD3 were used as controls for gating. The stained cells were measured on a Facscalibur (BD, Accuri C6) flow cytometer and analyzed by FlowJo_V10 software.

Software. All statistical analyses were performed on Origin (version 8.6), SPSS (version 22), Graphpad prism (version 7) or Excel 2016. Living image software (Version 4.5) was used to analyse bioluminescent and fluorescent images. Image J (Version 1.48h3) was used for fluorescence-image analysis. Transcriptomics data were analyzed online with I-Sanger Cloud Platform. FlowJo (Version 4.5) was used for flow cytometry analysis. Simca-P (Version 13.0) was used for metabolic analysis. HEML (Version 1.0) was used for heat map drawing.

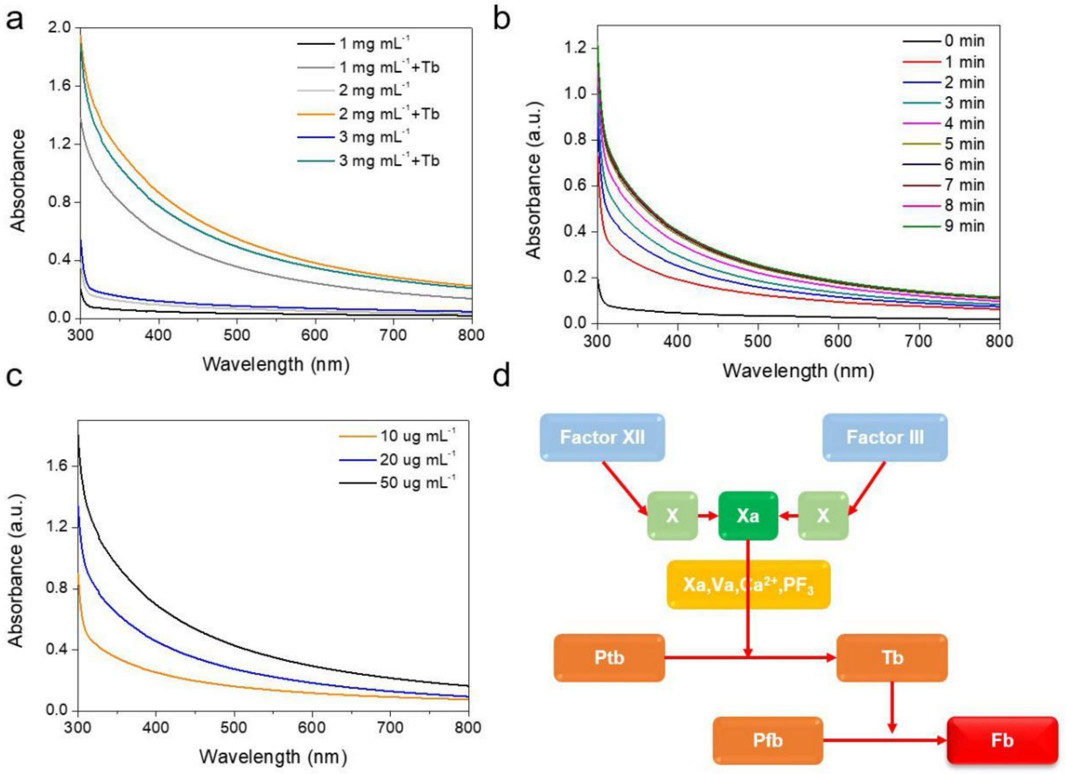

Supplementary Fig. 1 | Characterization and gelation of aECM. a, The UV-vis spectrum of fibrinogen at different concentration with and without addition of thrombin (40 μg mL^{-1}). With the addition of thrombin (Tb), the absorbance increases dramatically. **b,** The UV-vis spectrum of fibrinogen (1 mg mL^{-1}) with addition of thrombin changes over time. With time prolong, the absorbance increases. **c,** The UV-vis spectrum of fibrinogen (1 mg mL^{-1}) with addition of different concentration of thrombin. **d,** Schematic diagram of clotting mechanism. In response to bleeding, the prothrombin in plasma is proteolytically activated by thrombin followed by clots formulation through converting soluble fibrinogen into insoluble fibrin to stop the bleeding.

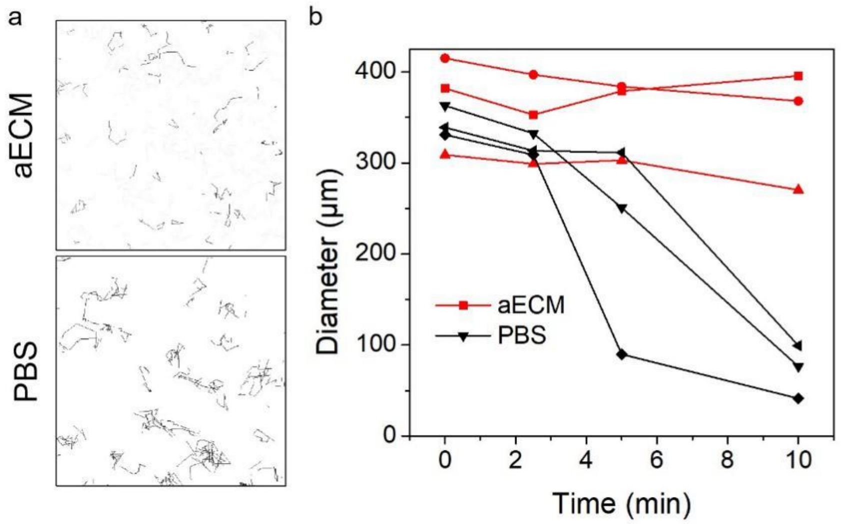

Supplementary Fig. 2 | The effect of aECM on preventing cell migration. a, The migratory path of the CT26 with or without aECM gelation. After inducing the *in vitro* gelation, the movement routes of CT26 cells within 11 h were analyzed with an Image J plug-in, MTrack2. A representative image of four biological replicates is shown. The size of the box is 300 μm × 300 μm. **b,** The change of CT26 MTS diameter with or without aECM treatment under shaken (300 rpm) for 10 min. Each set of experiments was repeated on three MTS.

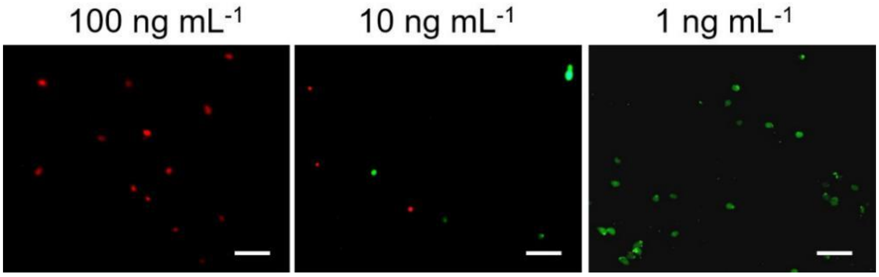

Supplementary Fig. 3 | Live/dead cell staining of aECM-Trail encapsulated CT26 cells. Fluorescence live/dead cell images of aECM-Trail treated CT26 cells with different concentrations of Trail (scale bar: 200 μm). Five images per group were taken.

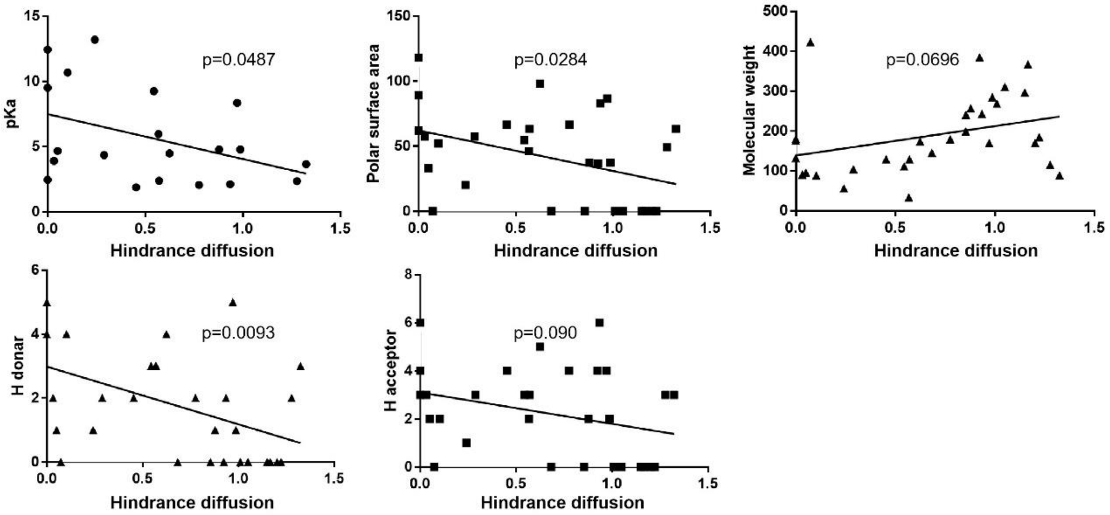

Supplementary Fig. 4 | Linear regression analysis of hindrance diffusion with various indexes. Variables of pKa, polar surface area and H donor number displayed a relative strong linear relationship with the hindrance diffusion. The p value was calculated with the linear regression. The physical properties of the metabolites were obtained from the scifinder database (https://www.cas.org/products/scifinder).

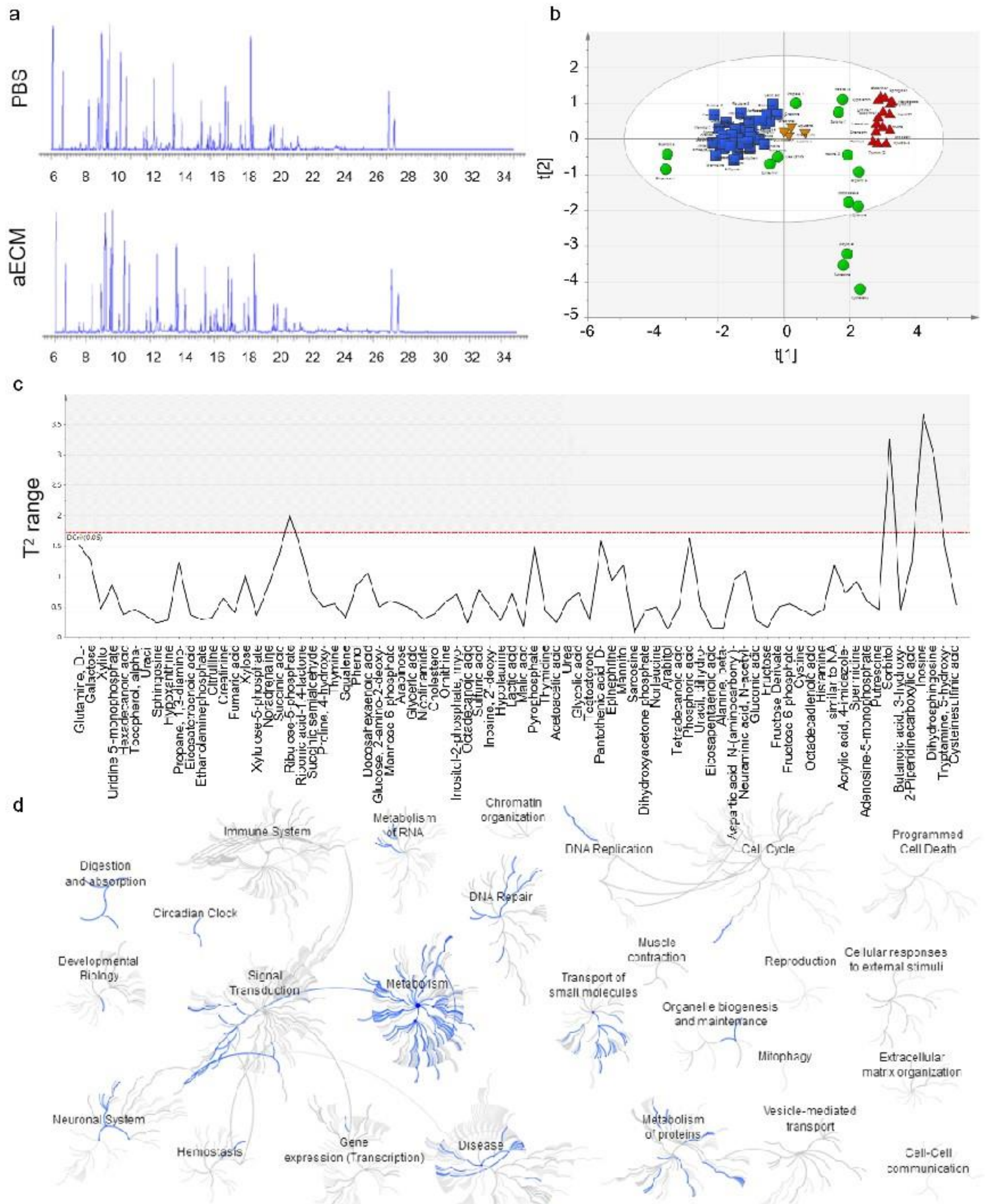

Supplementary Fig. 5 | Metabolomics study of aECM-treated CT26 tumors. a, Total ion chromatography of GC-MS analysis. **b,** Score scatter plot of various metabolites. These metabolites could be divided into two clusters (red and green). **c,** Hotelling's trace multivariate T test of all differential metabolites. T2 statistics with 95% and 99% confidence regions were calculated based on all differential metabolites. **d,**

The Reaction map shows the reactions annotated in Reactome. Up regulated pathway is highlighted in yellow, whereas down-regulated pathway is colored with blue. The reaction clusters of top-level processes are presented.

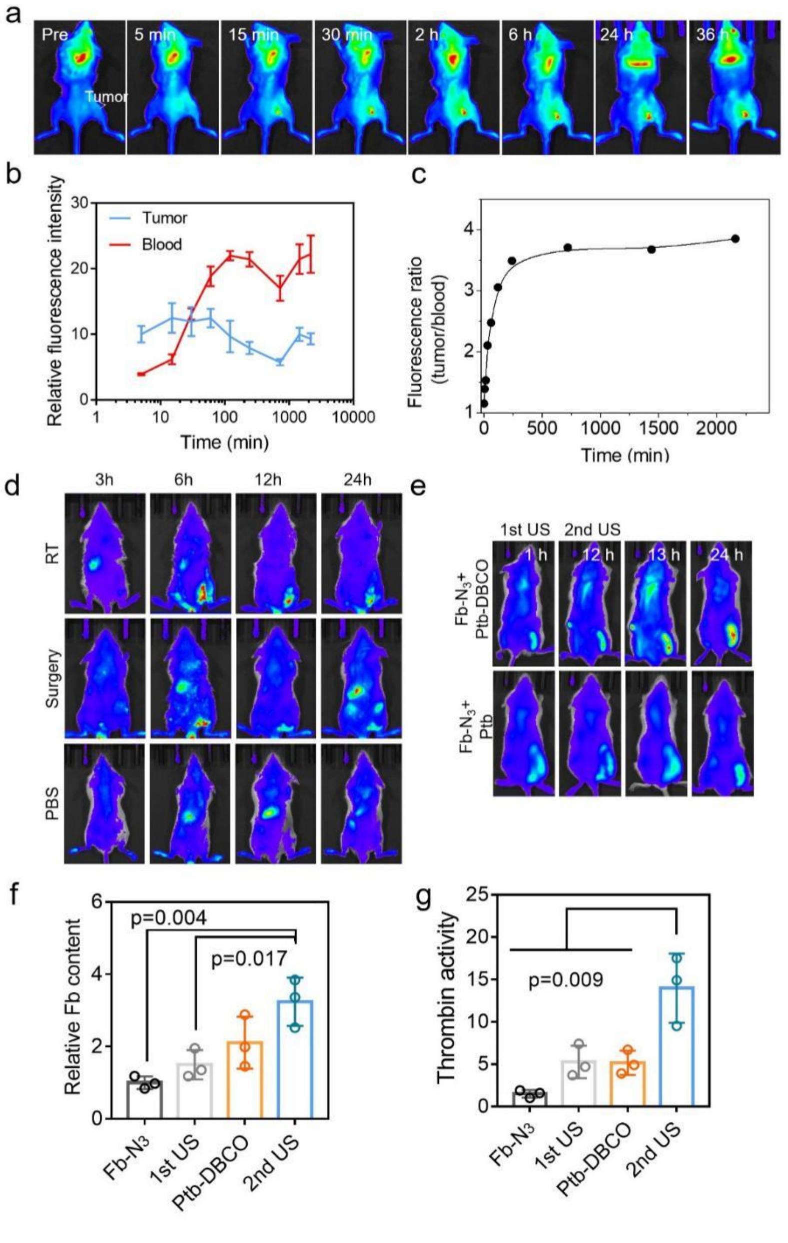

Supplementary Fig. 6 | *In vivo* accumulation of aECM. a, *In vivo* fluorescence images of aECM treated CT26 tumor bearing mice. The imaging was performed after US treatment at different time points. The *in vivo* fluorescence of Cy5 was used to determine the accumulation of Fb-N_3. Arrows denote tumor positions. A representative image of three biological replicates is shown. **b** and **c,** Semi-quantitative analysis of relative fluorescence intensity in tumor and blood of mice after injection with Cy5 labeled Fb-N_3 at different time points. Three biological replicates are shown. For

quantitative comparison, the regions of interest (ROI) were drawn over tumor and blood samples. The average signal for each area was measured. **d,** RT and surgery could also make micro-wound and induce the tumor-specific accumulation of Fb-N_3. The *in vivo* fluorescence of Cy7 was used to determine the accumulation of Fb-N_3. A representative image of three biological replicates is shown. **e,** *In vivo* fluorescence imaging for the capacity of Fb-N_3 in guiding tumor-specific accumulation of Ptb-DBCO. The biorthogonal strategy strongly promoted the accumulation of Fb-N_3 after the 2nd US treatment. A representative image of three biological replicates is shown. **f,** Fibrinogen ELISA kit for illustrating the tumor accumulation of Fb-N_3. Three biological replicates are shown. **g,** Thrombin activity fluorometric assay kit for indicating the activation of coagulation reaction. The thrombin activity was measured immediately after the treatment. Three biological replicates are shown. Significance between every two groups **(f, g)** was calculated using one-way ANOVA with Turkey post-hoc analysis. The mean values and S.D. are presented.

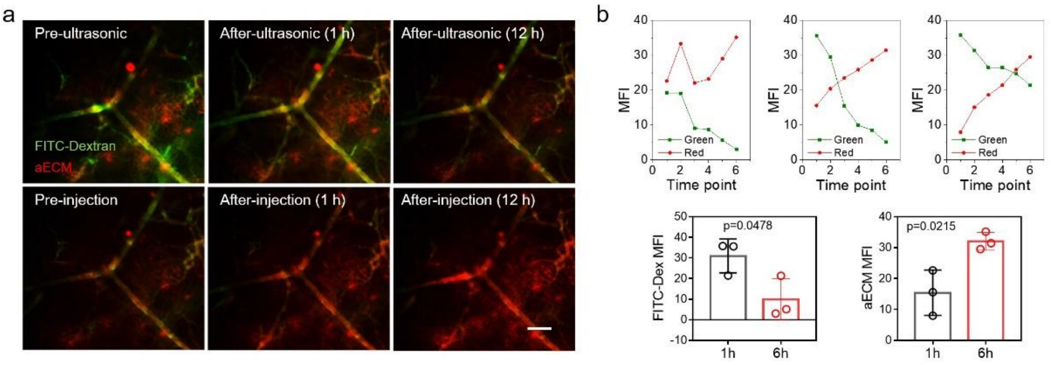

Supplementary Fig. 7 | Intravital fluorescence microscopy for observing the gelatinization of aECM in real time. a, The ear of mice was observed before and after (1 h and 12 h) the injection with rhodamine B isothiocyanate labeled fibrinogen. The US treatment prevented the transport of FITC-dextran and promoted the formation of aECM (scale bar: 50 μm). A representative image of three biological replicates is shown. **b,** The intravital fluorescence intensity of blood cells and aECM was quantified. Results from three independent experiments are shown. Significance between two groups was calculated using two tailed student's t test. The mean values and S.D. are presented.

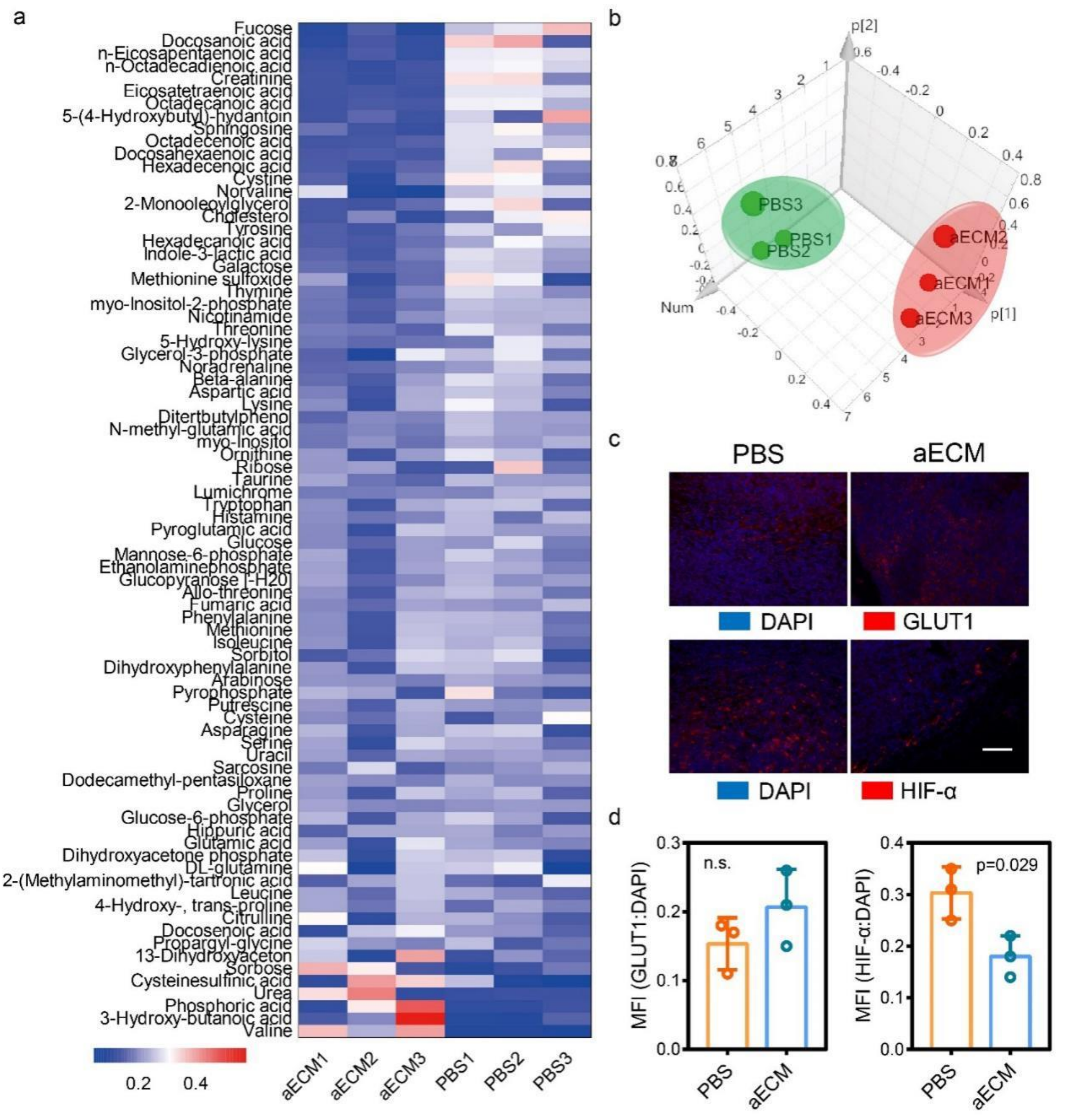

Supplementary Fig. 8 | The mechanism of aECM in 4T1 tumor. (**a**) Heat map representation and cluster analysis of metabolites in mice tumors after treated with aECM. Tumors from 4T1 tumor bearing mice were collected 24 h after inducing the aECM gelation. Three biological replicates are shown. (**b**) Principal component analysis of metabolites in mice 4T1 tumors before and after treated with aECM. (**c**) Immunofluorescence staining for GLUT1 and HIF-α expression in 4T1 tumors. 24 h after the *in vivo* gelation, mice tumors were collected. Tumor sections were stained for GLUT1 or HIF-α. Five images per group were taken (scale bar: 100 μm). (**d**)

Quantitative analysis of GLUT1 and HIF-1α expression in 4T1 tumors (n = 3 fields). The ratio of GLUT1 expression and nuclear fluorescence was calculated with Image J. Significance between two groups was calculated using two-tailed student's t-test (d). The mean values and S.D. are presented.

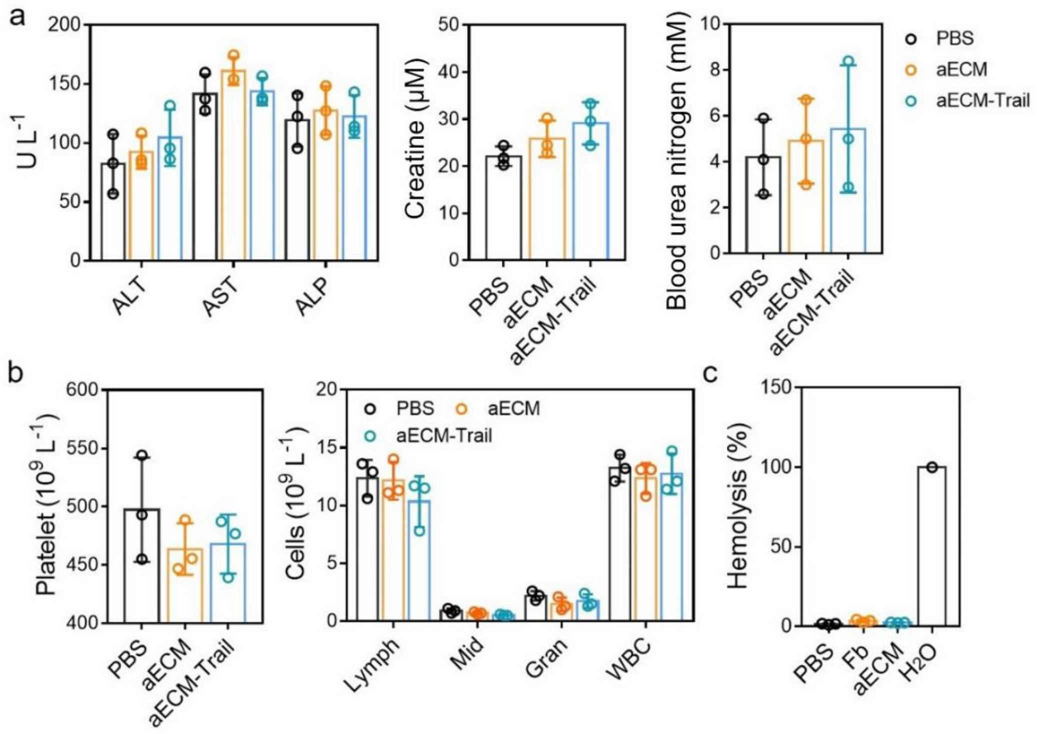

Supplementary Fig. 9 | *In vivo* biocompatibility study. a, Blood biochemistry data of liver and kidney function markers including alanine transaminase (ALT), aspartate transaminase (AST), alkaline phosphatase (ALP), blood urea nitrogen (BUN) and creatine (CRE). Three biological replicates are shown. **b,** Hematologic indexes of mice after treatment with aECM. Three biological replicates are shown. **c,** Hemolysis assay of aECM (n = 3). Complete lysis of red blood cells, induced by DI water was considered as a control (100% hemolysis). Three biological replicates are shown. Significance between two groups was calculated using two-tailed student's t-test. The mean values and S.D. are presented.

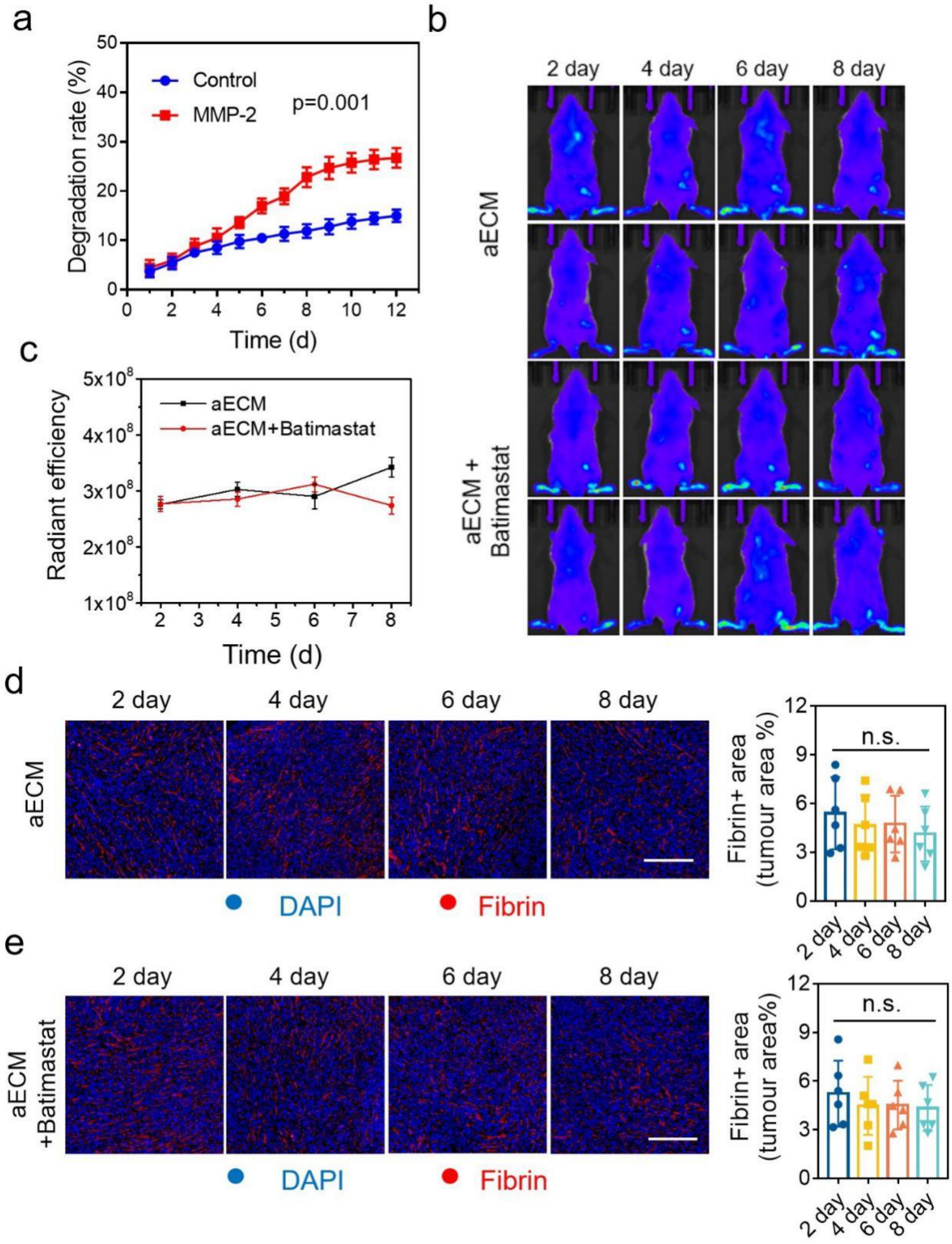

Supplementary Fig. 10 | The degradation of aECM. a, Cy7 release profiles of Cy7-labeled aECM. The aECM gel was immersed in MMP-2 (1.5 μg mL^{-1}) contained PBS buffer. aECM degradation was quantified by monitoring release of a fluorophore covalently bound to Fb. Three biological replicates are shown. **b,** Erosion of aECM in a subcutaneous CT26 tumor model was quantified by signal decay of a Cy7 dye

covalently bonded to Fb-N_3 before the aECM gel formation. A representative image of three biological replicates is shown. **c,** Quantitative study for the *in vivo* degradation of aECM gel. ROI was drawn over tumor, and the mean fluorescence intensity of Cy7 labeled Fb-N_3 was measured. The ROI area with same size and shape was used. Three biological replicates are shown. Significance between every two groups was calculated using two-tailed student's t-test. **d&e,** Immunofluorescence photos of aECM degradation in tumors. The fluorescence intensity was quantified using Image J (n = 6 fields, scale bar, 200 μm). Significance between every two groups was calculated by using one-way ANOVA with Tukey post-hoc analysis **(d, e)** or two tailed student's t test (**a**). The mean values and S.D. are presented.

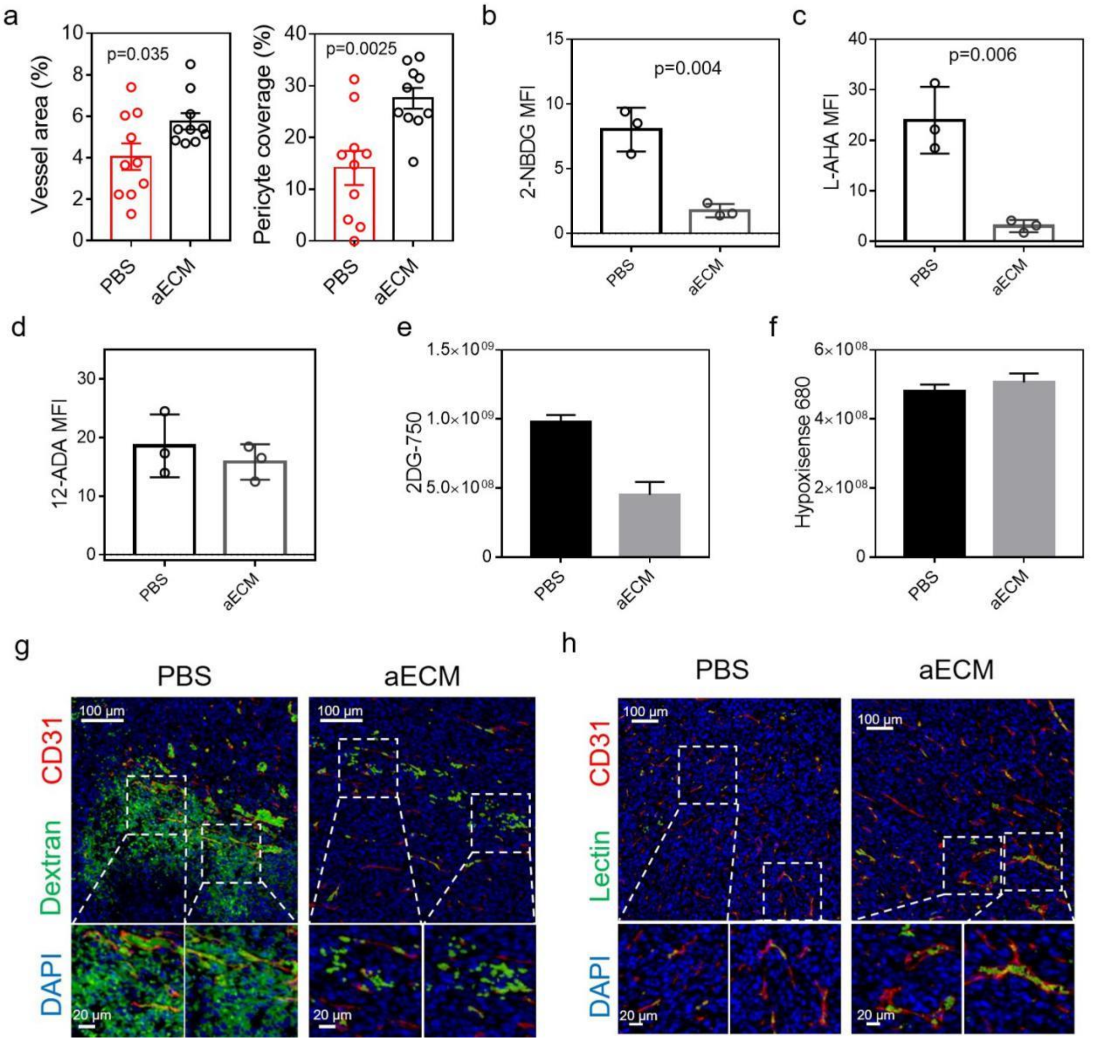

Supplementary Fig. 11 | Quantitative analysis for the *in vivo* effect of aECM. a, Vessel area and pericyte coverage in tumor tissues (n = 10 fields). **b,** Mean fluorescence intensity (MFI) of 2-NBDG fluorescence in tumors. Three biological replicates are shown. **c,** Mean fluorescence intensity (MFI) of L-AHA fluorescence in tumors. DBCO-Cy5 was used to mark L-AHA. Three biological replicates are shown. **d,** Mean fluorescence intensity (MFI) of 12-ADA fluorescence in tumors. DBCO-Cy5 was used to mark 12-ADA (n = 3 fields). **e,** Quantitative analysis for the intratumoral 2-DG 750 fluorescence. Two-dimensional regions of interest (ROI) circles were drawn to capture tumor fluorescence. The standard deviation is obtained with the ROI analysis of Living

image software of one image. **f,** Quantitative analysis for the intratumoral Hypoxisense 680 fluorescence. Two-dimensional regions of interest (ROI) circles were drawn to capture tumor fluorescence. The standard deviation is obtained with the ROI analysis of Living image software of one image. **g-h,** Immunofluorescence staining for lectin and dextran perfusion experiments. Three images per group were taken. The mean values and S.D. are presented. Significance between two groups was calculated by using two tailed student's t-test.

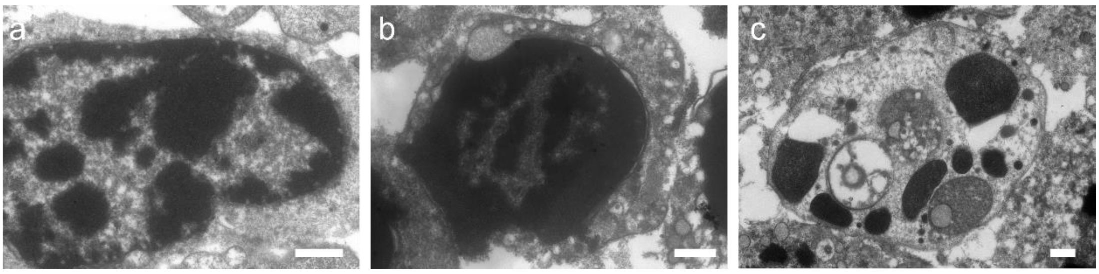

Supplementary Fig. 12 | TEM images of aECM treated tumor tissue. 48 h after the induction of aECM gelation, mice tumors were collected for TEM observation (n = 3 fields). Cell death related features, including cell disintegration (a), strong chromatin compaction in crescent shaped masses at the nuclear periphery (b), and apoptotic nuclear fission were found (c). This phenomenon revealed that the aECM treatment could trigger tumor cell death in mice (scale bar: 2 μm). The experiment was repeated twice independently with similar results.

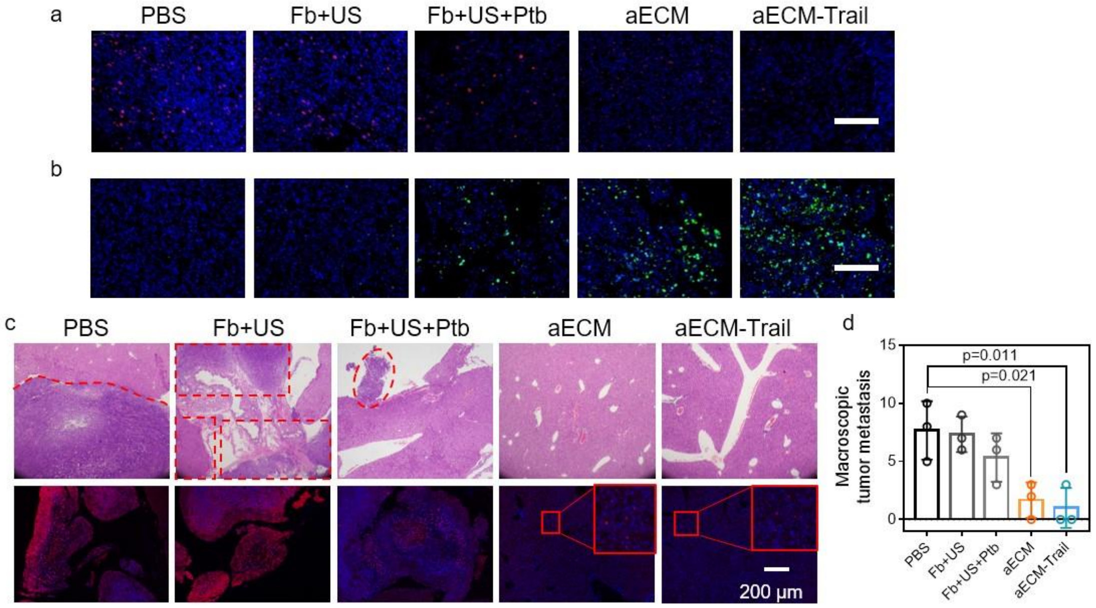

Supplementary Fig. 13 | *In vivo* anti-cancer effect of aECM in subcutaneous CT26 tumor-bearing mice. a and **b,** Immunofluorescence staining of Ki-67 and TUNEL of tumor after treatment with PBS, Fb + US, Fb + US + Ptb, aECM and aECM-Trail. aECM-Trail treated group showed a notably decreased Ki-67 fluorescence and increased TUNEL fluorescence, indicating inhibition of tumor proliferation after treatment. Three images per group were taken (scale bar: 200 μm). **c,** H&E staining and immunofluorescence staining of liver metastasis. Tumor volume curve of subcutaneous CT26 tumor (luciferase transfected) bearing mice after receiving various treatments. The treatment was started when the tumor volume reached to 100 mm^3 (n = 3 for each group). In H&E staining images, the macroscopic liver metastasis was circled. Firefly luciferase was also stained (red) to visualize the liver metastases. The infiltration of cancer cells in aECM or aECM-Trail treated tumor was enlarged. Three images per group were taken. **d,** The quantification of the metastatic burden. The number of macroscopic tumor metastasis the liver was counted. The size of the macro metastases

was similar (diameter ~ 1 μm). Three images per group were taken. Significance between every two groups was calculated by using one-way ANOVA with Tukey post-hoc analysis. The mean values and S.D. are presented.

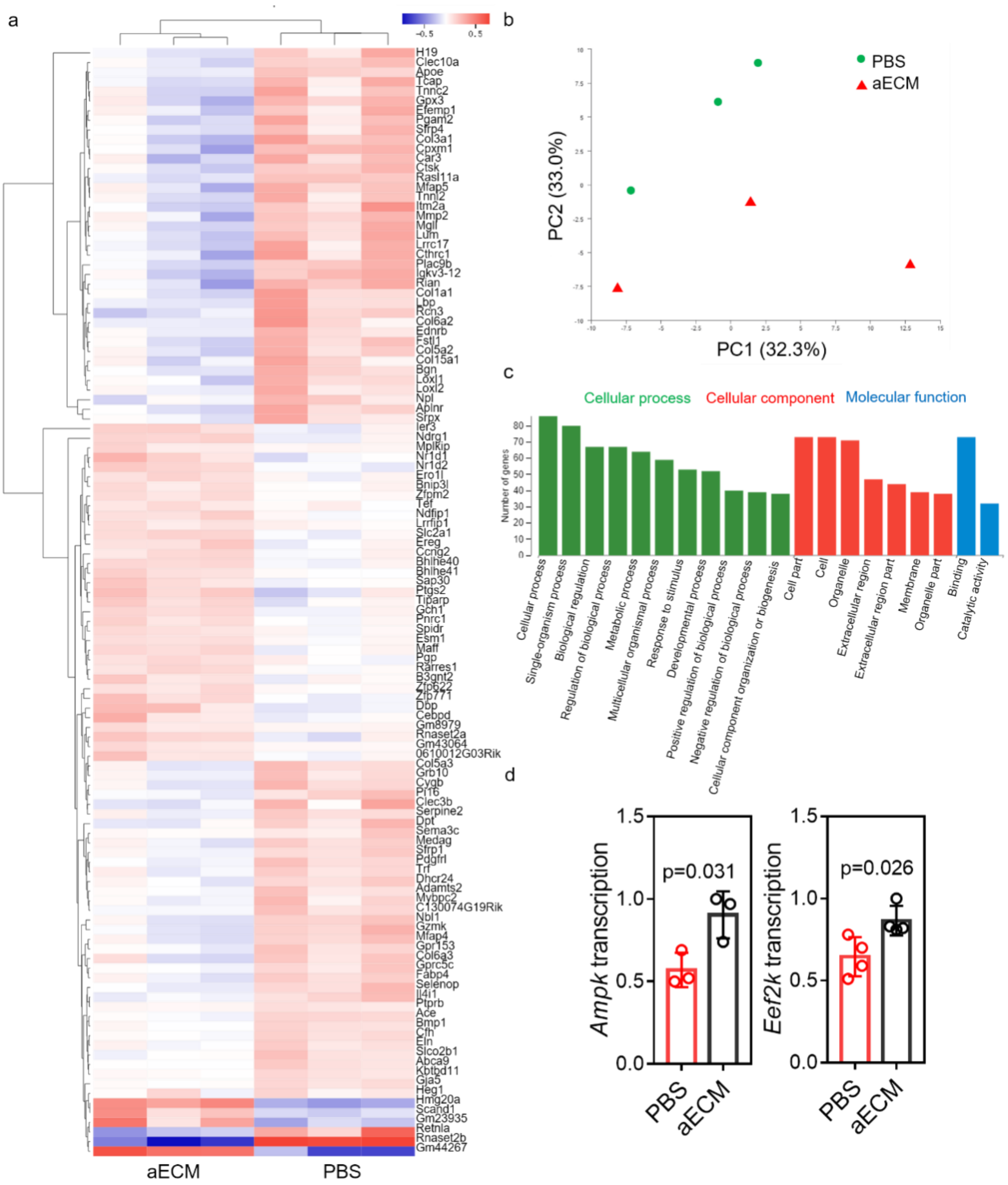

Supplementary Fig. 14 | Transcriptome study of aECM treated mice tumors. a, The cluster heat map of genes that were expressed differentially (change fold > 1.5, p value < 0.05) in PBS and aECM treated mice tumors. Cluster was identified to both genes and arrays. Three images per group were taken. **b**, Principal component analysis of transcriptome data. The scatter plots were generated representing each mouse in multivariate space by PC1 and PC2 score. **c**, Gene ontology (GO) pathway analysis of

the genes affected by the aECM treatment. The result of level 2 GO annotations analysis is presents. **d**, Analysis of two tumor starvation-associated genes, *Ampk* and *Eef2k* after aECM treatment. Three biological replicates are shown in *Ampk* and four biological replicates are shown in *Eef2k.* Significance between every two groups was calculated by using two-tailed student's t-test. The mean values and S.D. are presented.

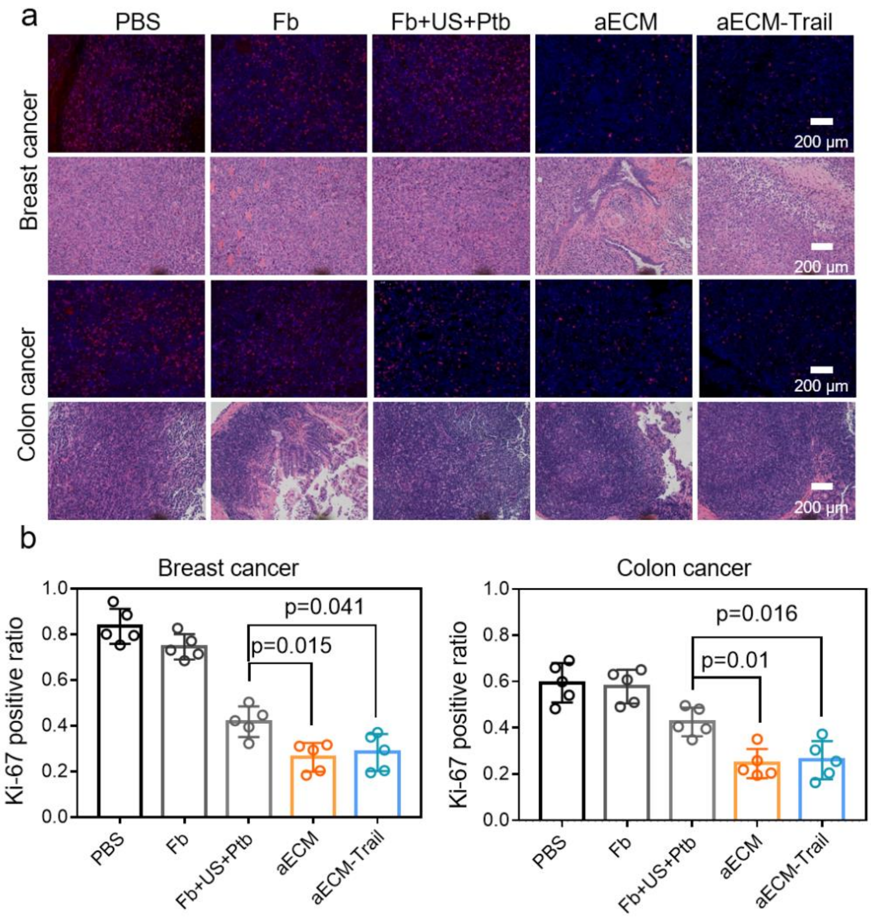

Supplementary Fig. 15 | *In vivo* anti-cancer effect of aECM in orthotopic tumor models. a, H&E and Ki-67 immunofluorescent staining of orthotopic breast cancer tissues and colon cancer tissues after treated with various agents as indicated. BALB/c mice-bearing orthotopic tumors were treated with various agents as indicated. Five images per group were taken. **b,** Quantification of Ki-67 staining in aECM-treated versus PBS-treated tumors at 3 days post-treatment (n = 5 fields). Significance between every two groups was calculated by using one-way ANOVA with Tukey post-hoc analysis. The mean values and S.D. are presented.

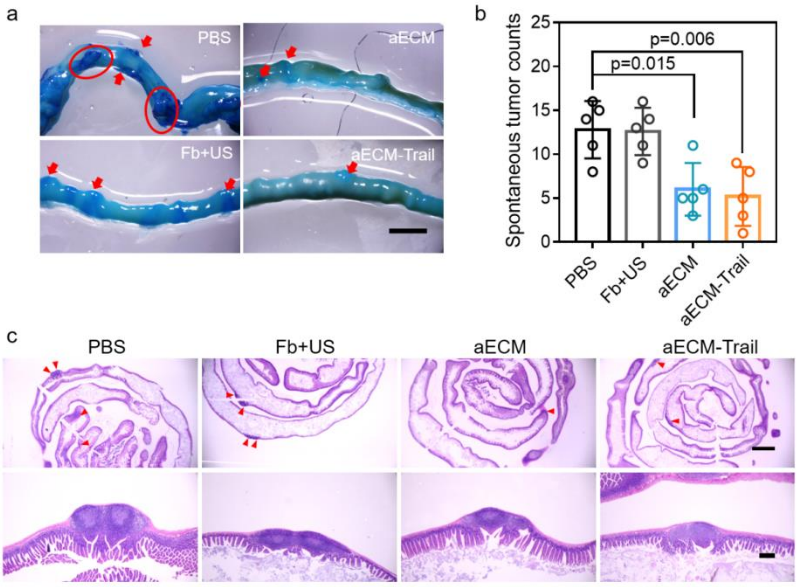

Supplementary Fig. 16 | The anti-cancer effect of aECM in *Apc*$^{Min/+}$ mice with spontaneous colon cancer. a, Microscopic images of spontaneous tumors in *Apc*$^{Min/+}$ mice after treated with various agents as indicated. Arrows and circles indicate the spontaneous tumors. A representative image of five biological replicates are shown (scale bar: 3 mm). **b**, Numbers of spontaneous tumors in *Apc*$^{Min/+}$ mice after treated with various agents. The tumor number was counted under stereomicroscope. Five biological replicates are shown. **c,** H&E staining of spontaneous tumors in *Apc*$^{Min/+}$ mice after treated with various agents. Three images per group were taken. Significance between every two groups was calculated by using one-way ANOVA with Tukey post-hoc analysis (scale bar: 2 mm, up; 200 μm, down). The mean values and S.D. are presented.

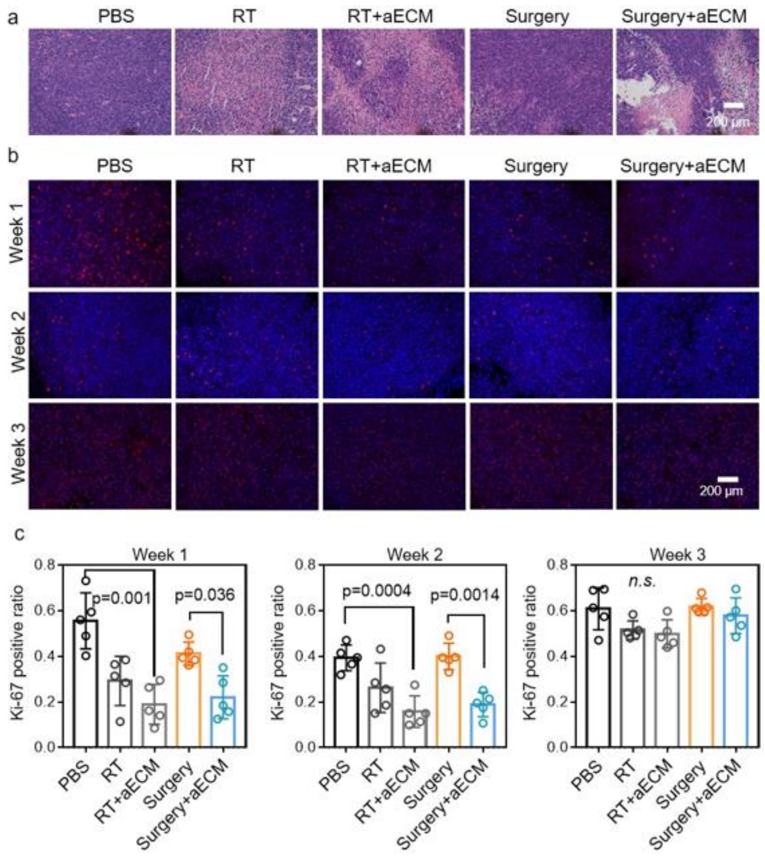

Supplementary Fig. 17 | *In vivo* anti-cancer effect of aECM and clinical therapies in subcutaneous CT26 tumor-bearing mice. a, H&E staining of colon cancer tissues after treated with various agents as indicated. The tumor samples were collected after 3-week of each therapy. Three images per group were taken. **b,** BALB/c mice-bearing subcutaneous CT-26 tumors were treated with various agents as indicated. At different time points after the treatments, mice were euthanized and tumors were embedded for sectioning. Tumor sections were stained for Ki-67. Five images per group were taken. **c,** Quantification (mean + SD) of Ki-67 staining in aECM-treated versus PBS-treated tumor at different time points post-treatment (n = 5 fields). Significance between every two groups was calculated by using one-way ANOVA with Tukey post-hoc analysis. The mean values and S.D. are presented.

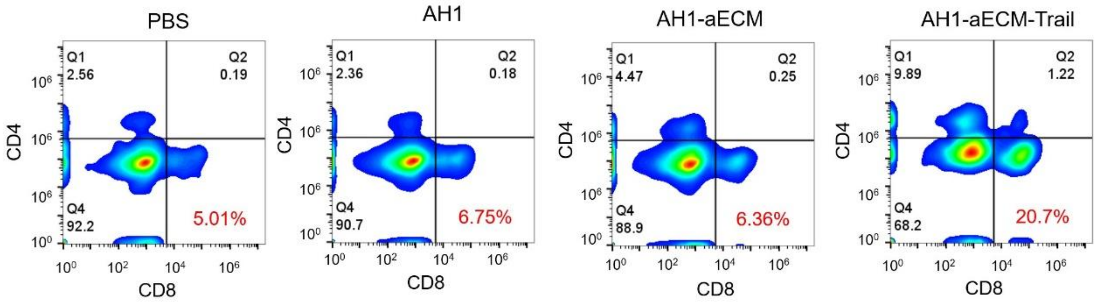

Supplementary Fig. 18 | Flow cytometry. Representative flow cytometric analysis images of $CD8^+CD4^-$ cells gating on $CD3^+$ lymphocytes in the tumor. Before staining, the cells were incubated with mouse anti-CD16/32 mAb to block non-specific Fc binding. Then, cells were stained with FITC anti-mouse CD3 Antibody, PE anti-mouse CD4 antibody, and APC anti-mouse CD8a antibody. Unstained cells and cells stained with CD3 were used as controls.
