# Supplementary material for: Controllable gelation of artificial extracellular matrix for altering mass transport and improving cancer therapies
Source: Nat Commun. 2020 Sep 30;11:4907. doi: 10.1038/s41467-020-18493-7 (PMC7527557; doi:10.1038/s41467-020-18493-7)
Supplement: Supplementary file 2 — Reporting Summary [file 41467_2020_18493_MOESM2_ESM.pdf]

nature research

Corresponding author(s): Xian-Zheng Zhang

Reporting Summary

Nature Research wishes to improve the reproducibility of the work that we publish. This form provides structure for consistency and transparency in reporting. For further information on Nature Research policies, see Authors & Referees and the Editorial Policy Checklist.

Statistical parameters

When statistical analyses are reported, confirm that the following items are present in the relevant location (e.g. figure legend, table legend, main text, or Methods section).

n/a	Confirmed	
	x	The exact sample size (n) for each experimental group/condition, given as a discrete number and unit of measurement
	x	An indication of whether measurements were taken from distinct samples or whether the same sample was measured repeatedly
	x	The statistical test(s) used AND whether they are one- or two-sided<br>*Only common tests should be described solely by name; describe more complex techniques in the Methods section.*
	x	A description of all covariates tested
	x	A description of any assumptions or corrections, such as tests of normality and adjustment for multiple comparisons
	x	A full description of the statistics including central tendency (e.g. means) or other basic estimates (e.g. regression coefficient) AND variation (e.g. standard deviation) or associated estimates of uncertainty (e.g. confidence intervals)
	x	For null hypothesis testing, the test statistic (e.g. F, t, r) with confidence intervals, effect sizes, degrees of freedom and P value noted<br>*Give P values as exact values whenever suitable.*
x		For Bayesian analysis, information on the choice of priors and Markov chain Monte Carlo settings
x		For hierarchical and complex designs, identification of the appropriate level for tests and full reporting of outcomes
x		Estimates of effect sizes (e.g. Cohen's d, Pearson's r), indicating how they were calculated
	x	Clearly defined error bars<br>*State explicitly what error bars represent (e.g. SD, SE, CI)*

Our web collection on statistics for biologists may be useful.

Software and code

Policy information about availability of computer code

Data collection	No software was used.
Data analysis	All statistical analyses were performed on Origin (version 8.6), SPSS (version 22), Graphpad prism (version 7) or Excel 2016. Living image software (Version 4.5) was used to analyse bioluminescent and fluorescent images. Image J (Version 1.48h3) was used for fluorescence-image analysis. Transcriptomics data were analyzed online with I-Sanger Cloud Platform. FlowJo (Version 4.5) was used for flow cytometry analysis. Simca-P (Version 13.0) was used for metabolic analysis. HEML (Version 1.0) was used for heat map drawing.

For manuscripts utilizing custom algorithms or software that are central to the research but not yet described in published literature, software must be made available to editors/reviewers upon request. We strongly encourage code deposition in a community repository (e.g. GitHub). See the Nature Research guidelines for submitting code & software for further information.

Data

Policy information about availability of data

All manuscripts must include a data availability statement. This statement should provide the following information, where applicable:

- Accession codes, unique identifiers, or web links for publicly available datasets
- A list of figures that have associated raw data
- A description of any restrictions on data availability

The source data underlying Figs. 1e, f, 2a-e, h, 3a–b, 4a, h, 5c, f, 6a, c, d, f, j, 7b, d, e and Supplementary Figs. 1, 2, 6-11, and 13-17 are provided as a Source Data file. All the relevant data are available from the authors upon reasonable request. The transcriptomic data are available at NCBI under Project PRJNA629444 [https://www.ncbi.nlm.nih.gov/Traces/study/?acc=PRJNA629444&o=acc_s%3Aa]. A reporting summary for this article is available as a Supplementary Information file.

Field-specific reporting

Please select the best fit for your research. If you are not sure, read the appropriate sections before making your selection.

☒ Life sciences ☐ Behavioural & social sciences ☐ Ecological, evolutionary & environmental sciences

For a reference copy of the document with all sections, see nature.com/authors/policies/ReportingSummary-flat.pdf

Life sciences study design

All studies must disclose on these points even when the disclosure is negative.

Sample size	No sample size calculation was performed. Sample sizes were determined according to previous experimental experience. The sample sizes of in vitro experiments refers to previously published literature (Nature Nanotechnology, 2019, 14, 89-97). The sample sizes of in vivo experiments refers to previously published literature (Nature Biomedical Engineering, 2018, 2, 611-621).
Data exclusions	No data were excluded.
Replication	All experimental data are given including replicates. Details of experimental replicates are given in the figure legends. All reported attempts at replication were successful.
Randomization	All samples/organisms were randomly allocated into experimental groups.
Blinding	Blinding was applied to all experiments.

Reporting for specific materials, systems and methods

Materials & experimental systems

n/a	Involved in the study	
☒	☐	Unique biological materials
☐	☒	Antibodies
☐	☒	Eukaryotic cell lines
☒	☐	Palaeontology
☐	☒	Animals and other organisms
☒	☐	Human research participants

Methods

n/a	Involved in the study	
☒	☐	ChIP-seq
☐	☒	Flow cytometry
☒	☐	MRI-based neuroimaging

Antibodies

Antibodies used	The primary antibodies for immonofluorescence were anti-Ki-67 rabbit monoclonal antibody (GB11030, dilution: 1:200) from Servicebio, anti-alpha smooth muscle actin antibody [E184] (ab32575, dilution: 1:200), anti-CD31 antibody [P2B1] (ab24590, dilution: 1:200), and anti-glucose transporter GLUT1 antibody [EPR3915] (ab115730, dilution: 1:200) from Abcam. Fibrinogen alpha chain polyclonal antibody (20645-1-AP, dilution: 1:100) was purchased from Proteintech. FITC anti-mouse CD3 antibody [17A2] (100203, 5 μg mL-1), PE anti-mouse CD4 antibody [GK1.5] (100407, 2.5 μg mL-1) , and APC anti-mouse CD8a antibody [53-6.71] (100711, 2.5 μg mL-1) were purchased from Biolegend.
Validation	All antibodies were validated by the commercial supplier. All validation statements can be found on the respective antibody website:<br>Anti-Ki-67 rabbit monoclonal antibody https://www.servicebio.cn/goodsdetail?id=1331<br>Anti-alpha smooth muscle actin antibody<br>https://www.abcam.com/alpha-smooth-muscle-actin-antibody-e184-ab32575.html<br>Fibrinogen alpha chain polyclonal antibody<br>https://www.ptgcn.com/Products/Fibrinogen-alpha-chain-Antibody-20645-1-AP.htm<br>Anti-glucose transporter GLUT1 antibody<br>https://www.abcam.com/glucose-transporter-glut1-antibody-epr3915-ab115730.html<br>Anti-CD31 antibody<br>https://www.abcam.com/cd31-antibody-p2b1-ab24590.html<br>PE anti-mouse CD4 antibody<br>https://www.biolegend.com/en-us/products/pe-Anti-mouse-cd4-Antibody-250<br>FITC anti-mouse CD3 antibody<br>https://www.biolegend.com/en-us/products/fitc-Anti-mouse-cd3-Antibody-45<br>APC anti-mouse CD8a antibody<br>https://www.biolegend.com/en-us/products/apc-anti-mouse-cd8a-antibody-150

Eukaryotic cell lines

Policy information about cell lines

Cell line source(s)	CT26 colon cancer cells, HT29 colon cancer cells, MCF-7 breast cancer cells and 4T1 breast cancer cells were all obtained from American type culture collection (ATCC).
Authentication	The cell lines were not authenticated.
Mycoplasma contamination	All cell lines were tested for mycoplasma contamination. No mycoplasma contamination was found.
Commonly misidentified lines<br>(See ICLAC register)	No commonly misidentified cell lines were used.

Animals and other organisms

Policy information about studies involving animals; ARRIVE guidelines recommended for reporting animal research

Laboratory animals	Six-week-old Balb/c female mice (18 ± 2g) and eight-week old male C57BL/6J-ApcMin/+ mice were used for study. Animals were housed in groups of 4-6 mice per individually ventilated cage in a 12 h light dark cycle (06:30-18:30 light; 18:30-06:30 dark), with constant room temperature (21 ± 1 °C) and relative humidity (40-60 %). Animals had access to food and water ad libitum.
Wild animals	The study did not involve wild animals studies.
Field-collected samples	The study did not involve filed-collection samples.

Flow Cytometry

Plots

Confirm that:

- [x] The axis labels state the marker and fluorochrome used (e.g. CD4-FITC).
- [x] The axis scales are clearly visible. Include numbers along axes only for bottom left plot of group (a 'group' is an analysis of identical markers).
- [x] All plots are contour plots with outliers or pseudocolor plots.
- [x] A numerical value for number of cells or percentage (with statistics) is provided.

Methodology

Sample preparation	Before staining, the cells were incubated with mouse anti-CD16/32 mAb to block non-specific Fc binding. Then, cells were stained with FITC anti-mouse CD3 Antibody, PE anti-mouse CD4 antibody, and APC anti-mouse CD8a antibody. Unstained cells and cells stained with CD3 were used as controls.
Instrument	Facscalibur (BD, Accuri C6)
Software	FlowJo_V10
Cell population abundance	No post-sort fractions were collected through the Flow cytometry.
Gating strategy	The gating strategy was detailly exhibited in Figure S18. CD8+CD4- cells were gating on CD3+ lymphocytes.

☒ Tick this box to confirm that a figure exemplifying the gating strategy is provided in the Supplementary Information.
